# Beyond Awareness: A Qualitative Assessment of Barriers and Facilitators to Implementing Metabolic Dysfunction–Associated Steatotic Liver Disease Care Pathways in Primary Care

**DOI:** 10.1016/j.gastha.2026.100991

**Published:** 2026-05-05

**Authors:** Stan Driessen, Leonard (Niels) D. Broekman, Anne de la Croix, Marianne C. Mak-Van der Vossen, Maarten E. Tushuizen, Otto R. Maarsingh, Adriaan (Onno) G. Holleboom

**Affiliations:** 1 Department of Vascular Medicine, Amsterdam UMC location University of Amsterdam, Amsterdam, the Netherlands; 2 Department of Gastroenterology and Hepatology, Amsterdam UMC location University of Amsterdam, Amsterdam, the Netherlands; 3 Department of General Practice/Family Medicine, Amsterdam UMC location University of Amsterdam, Amsterdam, the Netherlands; 4 Department of Gastroenterology and Hepatology, LUMC, Leiden University, Leiden, the Netherlands

**Keywords:** Metabolic Dysfunction–Associated Steatotic Liver Disease, Interview, General Practitioner, Care Innovation, Care Pathway

## Abstract

**Background and Aims:**

Health-care systems must adapt to a rising burden of metabolic dysfunction–associated steatotic liver disease (MASLD), yet implementation in primary care remains challenging. While existing strategies often target knowledge deficits, our study uniquely examines how cognitive, contextual, and experiential factors shape general practitioners’ (GPs) decision-making. This qualitative study explores GPs’ experiences with MASLD care and previous care innovations to identify barriers and facilitators to implementing MASLD care pathways in primary care.

**Methods:**

We conducted semistructured interviews with 15 Dutch GPs from diverse backgrounds, enhancing data richness through cointerviewing. Guided by an interpretivist approach, we applied inductive thematic analysis per Braun and Clarke. Maximum variation sampling was applied, and interviewing continued until sufficient information power was achieved for analytic sufficiency.

**Results:**

Findings were organized using a sliding scale framework: 1 consistent theme promoting innovation and 3 dynamic themes that can facilitate or impair innovation. GPs expressed strong intrinsic motivation for innovations, rooted in patient-centeredness and lifelong learning, yet continuously weighed this against identity-related, relational, and practical considerations. GPs stressed that primary care is not merely hospital care at a different place. They evaluate innovations pragmatically through a Bayesian lens, considering prior disease probability, test performance, and feasibility, combined with assessment of individual patient benefit. Key barriers included low perceived urgency for MASLD, driven by limited experiential knowledge and skepticism toward specialist-derived evidence. GPs perceived trust-based partnerships, organizational continuity, and contextual fit as critical.

**Conclusion:**

Implementing hepatology-derived innovations in primary care is complex and extends beyond financial incentives or educational interventions. Effective strategies require codesigned, context-sensitive, and practice-integrated approaches that align with GPs’ distinct logic and perspectives, including of those less engaged with MASLD.

## Introduction

As disease burden rises, adaptation of the health-care system is often necessary to optimize health care. A central component is changing physician behavior, which remains a widely recognized implementation challenge.^1^ The rising prevalence and severity of the steatotic liver diseases (SLDs), largely driven by the increasing incidence of metabolic dysfunction–associated steatotic liver disease (MASLD) and metabolic dysfunction–associated alcohol-related liver disease, bring an accompanying care organization conundrum, exemplifying these concepts. The current worldwide prevalence of MASLD is estimated at 31% of the adult population with substantial regional differences and is expected to increase over the coming years. Substantial regional variation in MASLD prevalence exists, ranging between 25% in Western Europe and 44% in Latin America, respectively.^2^ Metabolic dysfunction–associated steatohepatitis drives the advanced stages of fibrosis and cirrhosis, leading to substantial and gradually increasing health-care costs.^3^ Multiple national and international guidelines recommend noninvasive tests (NITs) for hepatic fibrosis as the current best practice for diagnosing and managing SLD.^4^^,^^5^ However, substantial discrepancies between SLD reference guidelines and real-world practice have been observed.^6^ This challenge extends beyond SLD, as adherence to established secondary prevention programs, such as those for diabetic retinopathy and chronic kidney disease, remains complex, particularly in primary care.^7^^,^^8^

In the case of MASLD, disconnections between guidelines and practice are often attributed to limited provider awareness and knowledge, prompting a predominant focus on education to improve adherence.^9^, ^10^, ^11^ However, broader change management frameworks highlight cognitive, attitudinal, and contextual domains that remain underexplored in SLD literature yet might be crucial in sustainable implementation of care pathways.^12^ Earlier qualitative studies have emphasized general perceptions of NITs and MASLD care, while overlooking experiential and tacit knowledge: the lived, practice-based insights crucial to understanding complex care dynamics.^13^ In MASLD, this poses a particular challenge, as limited awareness of the disease burden restricts opportunities to develop experiential understanding.^14^ Yet, capturing the perspectives of a broad range of clinicians, including those with limited MASLD experience, is essential to explain suboptimal uptake of MASLD innovations. Experiential insight is often lacking, especially in primary care, where advanced MASLD is less prevalent than in specialized settings.^2^

From a Bayesian perspective, clinical decision-making involves updating disease probability based on prior prevalence and test performance. This framework is vital in illustrating the distinction between primary and specialized care, especially in diagnostic use and interpretation of test results.^15^ In primary care, the lower prevalence of advanced MASLD necessitates a distinct approach, as general practitioners (GPs) must interpret inconclusive evidence within broader differentials and with different test performance. Paradoxically, despite encountering fewer advanced MASLD cases, GPs remain central to screening and prevention, as they manage the majority of at-risk patients through cardiometabolic care programs. Knowledge transfer from specialist to primary care is often gradual, shaped by differences in priorities, context, and perceived relevance. Current MASLD guidelines originated in specialist settings, but dedicated primary care guidance is still lacking, and GPs remain underrepresented in related research.^16^^,^^17^ Recognizing this stepwise nature of knowledge transfer highlights the need for earlier, proactive primary care engagement in addressing emerging burdens like MASLD.

Although knowledge deficits have been identified as barriers to MASLD care pathway implementation, critical gaps remain in understanding how primary care professionals experience, interpret, and navigate recommendations in routine practice. This study explored GPs’ experiences with MASLD and prior health-care innovations, in particular secondary prevention programs, to inform strategies for MASLD screening and management. In this qualitative study, we investigated barriers and facilitators to implementing MASLD fibrosis care pathways in Dutch primary care, a setting well suited given GPs’ gatekeeping role^18^ and established cardiometabolic care infrastructure.^19^ While MASLD is the focal point of this study, the insights into GPs’ reasoning, innovation uptake, and contextual navigation may also inform implementation of other secondary prevention pathways in primary care.

## Methods

### Study Design

We conducted a qualitative study using semistructured interviews, maintaining an interpretivist approach, which views knowledge as shaped by social interaction and open to multiple interpretations.^20^ Consequently, we acknowledge that our perspectives and experiences influence data collection and interpretation. Our interdisciplinary team includes a hepatologist (M.T.), an endocrinologist/vascular medicine specialist with expertise in MASLD (O.H.), 2 GPs, 1 focused on health-care continuity (O.M.), and 1 on qualitative research and professional identity in medical education (M.M.), a qualitative health and education researcher with a background in linguistics and ample experience in supervising qualitative research projects and teaching courses on qualitative research (A.C.), and 2 MD-PhD candidates (N.B. and S.D.) studying MASLD-fibrosis care pathways implementation, with interests in health care’s socioeconomic context and interdisciplinary collaboration. The MD-PhD candidates were trained, coached, and supervised in qualitative research by 1 GP and the qualitative health and education researcher, and completed formal courses. Our diverse backgrounds fostered rich discussion and nuanced interpretation of the health-care context.

### Study Setting

In the Dutch health-care system, GPs are the first point of contact for most medical concerns and function as gatekeepers, providing personalized referrals to specialized care.^18^ Primary care practices are typically small and independent, closely collaborating with physiotherapists, pharmacists, and neighboring GPs, either informally or through regional primary care organizations. Cardiovascular risk management is often delivered by practice nurses under GP supervision. An extensive description of the Dutch health-care system is provided in Supplementary Material 1.

### Sampling of Participants

We used maximum variation sampling to recruit GPs with diverse backgrounds, including those with and without roles in management, innovation, or guideline development; varying experience with NITs; and differing interests in type 2 diabetes, cardiovascular risk management, and lifestyle medicine.^21^ We also ensured variation in practice type (solo, group, multidisciplinary), location (urban/rural), age, and gender to capture a broad range of perspectives. Participants were identified via snowball sampling and primary care organization websites, then contacted directly. Twenty-nine GPs were contacted in total, and interviews were scheduled within several weeks.

The study followed the Declaration of Helsinki, was approved by the Amsterdam UMC Ethics Committee and exempted from further review (2025.0321), followed institutional and General Data Protection Regulation guidelines, and obtained audio-recorded informed consent. Data were deidentified and handled per Amsterdam UMC Public Health research code.

### Development of Interview Guide

We developed an interview guide to explore barriers and facilitators for implementing MASLD fibrosis care pathways in Dutch primary care, informed by literature on GPs’ MASLD knowledge, referral patterns, and guideline-practice gaps.^6^^,^^14^^,^^22^ The initial guide covered 4 domains: MASLD knowledge and vision, diagnostic methods, implementation, and health-care organization. After 3 pilot interviews (conducted by S.D.), the guide was refined to 2 domains: experiences and approaches to MASLD in daily practice and experiences with past health-care innovations and their relevance to MASLD. After observing limited MASLD-specific experiential knowledge in the pilots, we broadened the scope to include GPs’ experiences with innovations for other diseases. Emphasis during interviews varied depending on GPs’ MASLD experience, innovation background, and previous roles. The final interview guide is provided in Supplementary Material 2.

### Data Collection

Between January 2024 and May 2025, we conducted 15 semistructured interviews with GPs, either in person or via video call, with all participants providing recorded consent. To become familiar with the interview guide, S.D. and N.B. each conducted 1 solo interview, followed by a joint interview to align styles. As cointerviewing improved probing and depth, it was continued with alternating leader roles.^23^ Interviews lasted 34–55 minutes (median = 42), and field notes were written during each interview. All interviews were recorded and transcribed verbatim. Data interpretation was discussed in team meetings, and data collection was concluded based on sample diversity and analytic sufficiency.^24^ We applied the concept of information power to assess when sufficient depth and variation had been achieved.^25^ Details are provided in Supplementary Material 3.

### Data Analysis

We conducted an inductive thematic analysis following Braun and Clarke’s 6-phase framework.^20^ Data analysis began after the first interview and proceeded iteratively alongside data collection. To ensure trustworthiness and rigor, an audit trail was kept, documenting decisions on codebook development, theme refinement, and reflexive discussions. S.D. coded all transcripts inductively. S.D. and N.B. selected 3 context-rich interviews that were independently coded by N.B., after which code trees were discussed and merged. Thereafter, N.B. coded 1 in every 3 interviews, with S.D. consulting N.B. on new codes or patterns.^26^ Coding was done in MAXQDA 2022, and a native English speaker with Dutch fluency checked translations of selected quotes. Findings were organized around key semantic topics, capturing how participants conceptualized MASLD and previous care innovations. N.B. and S.D. regularly discussed theme development with each other and the broader team, focusing on rich and overlapping codes. The written analysis was cross-checked against codes and excerpts to ensure accuracy and representativeness, supporting a nuanced interpretation and avoiding overgeneralization. Final manuscript drafts were discussed by all authors to enhance confirmability and ensure alignment with the data.^27^

## Results

Our findings are based on interviews with 15 GPs with varying backgrounds. Seven were women, 5 were involved in national GP societies/organizations, 3 in regional organizations, 5 in MASLD initiatives, and both urban and rural areas were represented (Table 1).

**Table 1:** Descriptive Information About Participating General Practitioners

Characteristics	n (%, unless specified other)
Gender	
Female	7 (46.7%)
Male	8 (53.3%)
Age	
Median (range)	52 (36–65)
Practice type^a^^,^^b^	
Solo	4 (26.7%)
Duo	1 (6.7%)
Solo+	2 (13.3%)
Small group	2 (13.3%)
Medium-large group^f^	4 (26.7%)
Nonpracticing	2 (13.3%)
Patient population	
Median (range)	5100 (2000–13,000)
External activities next to regular clinical practice	
Involved in national society^c^	5 (33.3%)
Involved in regional society	3 (20.0%)
Involved in MASLD initiatives^d^	5 (33.3%)
Academic	3 (20.0%)
Area^e^	
Urban	8 (53.3%)
Suburban	5 (33.3%)
Rural	2 (13.3%)

a A distinction is made between GPs that practice together, under the same name, in a fully integrated group practice, and GPs that practice in a solo practice (here named “Solo+,” or “HOED” in Dutch), but under the same roof with several other solo practices, thereby sharing equipment, space, staff, etc.

b Small group practices are defined as practices with up to 4 permanently practicing GPs, while medium-large group practices are defined as practices with 5 or more permanently practices GPs.

c Dutch College of General Practitioners (NHG) or National Association of General Practitioners (Landelijke Huisartsen Vereniging).

d (Care pathway) studies or outreach endeavors.

e Urban, ≥1500 inhabitants/km^2^; suburban, 500/km^2^–1500/km^2^; rural, <500/km^2^.

f One of the GPs that practices in a group practice is practice owner as well.

We structured our findings using a sliding scale framework illustrating the continuous balancing act that GPs face when considering innovation (Figure). We describe 1 consistent theme that universally promotes change, and 3 dynamic themes that can either facilitate or hinder change depending on context. We begin with the consistent theme: The GPs’ strong willingness for change, and explore its underlying drivers. The 3 dynamic themes are categorized according to identity-related, social, and practical dimensions, while acknowledging their complexity and interrelatedness. Supporting quotes are presented in-text or provided in Table 2 and referenced to using quote-specific denotations.

**Figure:**
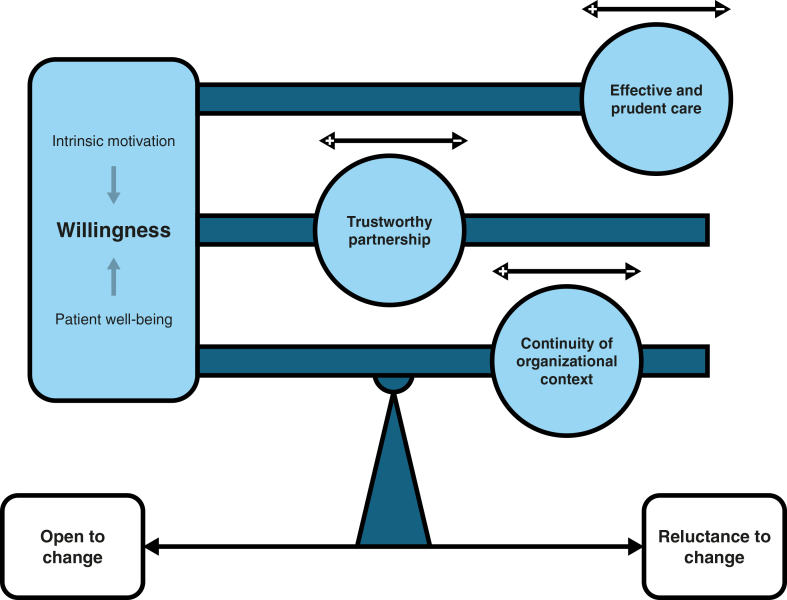
Conceptual model of innovation adoption in MASLD primary care: a dynamic balance framework. This figure visualizes the overarching and dynamic themes identified in our analysis, using the metaphor of sliding scales. When the scales tip to the left, the GP is inclined to adopt an innovation; when they tip to the right, the GP shows reluctance. The balance reflects how various factors weigh into the decision-making process. On one side, GPs' consistent willingness to change, driven by intrinsic motivation and commitment to patient well-being, serves as a strong enabler of adoption. This willingness is balanced against 3 dynamic and context-sensitive dimensions that can either facilitate or hinder innovation: the perceived alignment with effective and prudent care, the presence of a close and trustworthy partnership with other providers, and the continuity of the organizational context. Each of these themes acts as a fulcrum that can tip the decision toward openness or reluctance, reflecting the complex and multidimensional, ongoing trade-offs in general practice.

**Table 2:** Interview Excerpts Supporting Identified Themes

Theme	Supporting evidence
Willingness for change	**Intrinsic motivation** “Another key factor is attitude right? Specifically, that GPs are open to change, this differs greatly among them.” (9.1) “But it does motivate to, well, have consultations more often and it is also just informative, so you see that people tend to do it more often… so this learning really does have an effect.” (12.1) **Trade-off** “And you know, in practice you can look at things like: do you have enough people? Do you see the potential? Are you not going to make a loss? Because of course… I mean, we're entrepreneurs too.” (14.1)
Primary care is not just hospital care at a different place; the importance of effective and prudent care	**Guardians of effective and prudent care** “I now feel that from secondary care it’s more easily said, “that can also be done by the GP,” while I think some people are actually quite complex, and I do want to have said that I really believe that kind of care belongs in secondary care.” (5.1) “So, we’re in consultations with patients all day, and if you ask: who wouldn’t you have wanted to see? The answer is zero, because every patient who sits in front of you has a question, and you want to answer that question. Right? That’s our role.” (4.1) “I do see GPs very much as, what do you call that? Thermometers for this kind of public health developments… So that kind of development small or big, social or medical. You do notice that quite well as a GP.” (11.1) **Uncertainty, gut feeling, and guidelines** “And then you have the 10% who are the teacher’s pets. They're always ahead of the curve, they know all the guidelines, and they’re also willing to try something new. But they’re the exception. The other 80% just go about their work, some do well, some better, some less so. That’s roughly how you should see it. And yes, there will definitely be doctors who adopt this more quickly. But 80% will just look at the guideline.” (4.2) **Few experience with MASLD and its clinical consequences** “If you can’t present a strong case for it, many people will just think: I’ve got plenty of other things with stronger evidence, good luck with that!” (6.1)
Trustworthy partnership between specialist care and primary care	**Collaboration from the outset** “But in the past, the idea was that the hospital was better, right? Whereas we think: well, it can be done just fine here as well.” (5.2) **Sense of dual partnership, on an equal level** “Usually you will have a project that doesn't have support then, and if you don't have support, then that whole project will flop.” (14.2) “You need us, we need you and from that intrinsic motivation it should be: we are partners in crime and how can we help each other?” (4.3) “You have to look at it from a position of equality: how can we best serve the patient? It’s great if we take on certain tasks, but we also need the assurance that a patient can easily return if things don’t go well. So, having a good opportunity for consultation is essential.”(12.2) **Desire for lenient cooperation** “So most general practitioners are so committed to their patients, that they allow that to happen every time.” (2.1) **Informal nature of partnership** “And then the gastroenterologist says: “Can’t you do a calprotectin test?” And this happens quite often of course, that after discussing it a few times, you think: well, I’ll just do it myself now.” (11.2) “Or they see an example somewhere else and think: well, we want that to.” (12.3) **Centralization and the value of the mandate** “And they also have people who can sell that well, well, to the GP practices. And the regional organisation already has a certain goodwill in it, so that's also convenient. And otherwise, because you have to run it through a separate stream and that's just not feasible.” (13.1) “My first question is, you talk about implementation: what exactly is implementation?… Because when I sit down with the Ministry of Health, they’re talking about something completely different when it comes to implementation, and when I sit down with researchers, it’s also something entirely different; namely, getting it into the guideline… And the Ministry of Health, yes, what they really want is for people to become healthier, and above all, for it to cost less.” (8.1)
Continuity of organizational context	**Reimbursement** “And it's really not much, just several euros or so per digital consultation, and it does take quite a bit of time, so well, it's basically just cost-covering, but still it is motivating.” (12.4) **(Disconnect in perception of) workload** “The extra work you have to do has to be worth the reward. And sometimes, you know… GPs generally have enough, they earn enough. You don’t hear many GPs complaining about their income, but you do hear them complain about the workload” (9.2) “So this system changes a little every few years. And that’s quite frustrating because you can’t make any long-term plans.” (7.1) **Low complexity, (due to) integration in existing workflows** “You see, the lab is offering it again in our village, that wasn’t the case for a while, but now they’re doing it again. Patients no longer have to go to Groningen for it either. Well, now that you mention it, every barrier really is gone… And now it’s all just being handed to me on a silver platter.” (12.5) **Individualism, pragmatism, and fluidness of feasibility** “You can figure out for yourself how to do it. That’s also the fun part of my job.” (14.3)

### Overarching Consistent Theme: Willingness for Change

At the same time, I still believe that if it can be done in primary care, then it should be done in primary care… So, if you, as a patient, don’t need to go to secondary care because your GP has safely ruled out that it's necessary, well that’s amazing right? (Participant 8)

As an overarching theme, GPs consistently express a strong willingness to engage in care transformation, including adopting new tasks. This motivation is largely driven by their commitment to patient well-being. As participant 4 stated:

Look: doctors, all doctors, want the best for the patient sitting in front of them.

GPs also express an urgency for lifelong learning, fueling their interest in new health-care approaches (12.1). However, intrinsic motivation varies by personal preferences, interests, and propensity to adopt innovations (9.1).

GPs continuously balance this willingness to innovate with external constraints, resulting in a dynamic trade-off between openness and reluctance, as participant 12 noted: “Yes, because naturally you want to serve your patients well… So I am very willing to do that, but I do need the space and the opportunity to do so. Also, if for example, I can’t hire extra staff because there simply isn’t funding for it, then at some point it’s obviously going to cause friction.”

Dutch GP practices range mostly from small group practices to solo practices, with GPs retaining substantial autonomy and financial responsibility. This dual role, both health-care providers and entrepreneurs, results in careful cost-benefit considerations and underscores the importance of continuity (quote 14.1).

### Dynamic Theme 1: Primary Care Is Not Merely Hospital Care at a Different Place: The Importance of Effective and Prudent Care

One of the things I always tell residents, is to keep patients out of the clutches of a specialist. (Participant 3)

GPs view themselves as guardians of effective and prudent care, and their reluctance toward certain innovations often stems from differing interpretations with other health-care actors on what exactly this entails. Many described how past innovations have been hindered by discrepancies between perspectives of GPs and specialists on the necessity of innovations (5.1).

Discrepancies often concern the level of evidence required and expected clinical impact. GPs prefer primary care-derived evidence essential before altering practices, rather than extrapolating data from specialist care settings, as participant 6 explained:

You have to be able to convince GPs that it is also relevant and effective to do this in a primary care population. And as long as you can't do that, a lot of GPs, who are somewhat conservative by nature, for good reasons, that is also our role in the system, will not take action on it very strongly.

This reflects the distinct role of GPs in the health-care system. With most patient contacts occurring in primary care, interventions at this level significantly impact overall health-care costs. Subsequently, clinical relevance and efficiency are central to their decisions, as illustrated by participant 9:

So first, there is the scientific question: does this screening help? Because we are ex-tre-me-ly critical about screening, right? Since it concerns a very large group.

This aligns with the GPs’ holistic perspective, which prioritizes individual patient well-being over disease-focused care (4.1). GPs consider the full care process, including aspects beyond their control, and place high value on patient motivation and engagement. GPs may voice concerns about processes beyond their control, reflecting a broader sense of social responsibility, seeing themselves as indicators of societal disease trends (11.1).

The GP’s distinct role also shapes their approach to diagnostic uncertainty. GPs often rely on experience-based gut feeling in decision-making and tend to thrive in such contexts, as participant 14 illustrated:

You know, sometimes, all that us GPs really have are our hands and eyes, and a bag with a few other tools. And that’s all we can do.

This ostensibly intuitive approach is contrasted by the high value GPs place on national GP guidelines or regional agreements as a shared reference point within their otherwise individualized practice environment (4.2).

This tension between intuitive practice and guideline reliance may explain why GPs often viewed uncertainty reducing innovations as successful. Working in individualistic settings with autonomous, complex decision-making, GPs rely on guidelines or regional agreements as a supportive framework. In the context of MASLD, care pathways involving NITs may increase their sense of certainty and control.

However, GPs describe urgency for change as arising only when they personally perceive its necessity, as participant 3 noted:

Yes well first, I think there has to be a need! You have to feel that too, that need, otherwise you'll still be stuck in your old…

Of note, GPs do not yet experience MASLD as a pressing issue. Most responded “no” or “maybe a few” when asked if they had referred or managed patients with advanced MASLD fibrosis or cirrhosis. Whether or not this reflects true prevalence, it may contribute to different perceptions of the disease burden between primary and specialist care. In the context of MASLD, this may pose a barrier, as implementation must compete for attention and resources with other priorities across the broad scope of primary care (6.1).

### Dynamic Theme 2: Trustworthy Partnership Between Specialist Care and Primary Care

Why that became successful? That's because it was conceived together with GPs and specialists. (Participant 6)

While several factors contribute to strong partnerships, their effectiveness ultimately stems from trust.

GPs consistently noted that earlier innovations succeeded through close transmural collaborations from the outset. When excluded early on, they feel undervalued or disempowered, and innovations often fail to align with daily practice.

Although several participants raised concerns regarding care and workload substitution, most expressed a more nuanced view.

GPs strongly support keeping patients within primary care when appropriate and believe clearly defined problems are best managed there (5.2). The core concern is not task delegation itself but exclusion from preparatory decision-making. Patients are often “dumped,” or care tasks shift from specialized to primary care without prior consultation, as described by participant 2:

That's kind of the frustrated answer, but that's kind of what it comes down to. If you get the feeling as a primary care GP that you are carrying it together, then it becomes something very different.

Such lack of communication erodes trust and fosters resistance (14.2). As a result, GPs often instinctually decide whether to engage in innovations, partly based on personal trust in the innovation’s initiator, as underlined by participant 10:

And a project like that doesn’t really gain traction. You end up with people digging in their heels.

Additionally, GPs view health care as a team effort and treasure partnership (4.3, 12.2).

While they play a central role, this also makes them vulnerable, as they are often held ultimately responsible for the patient. This responsibility can lead them to reluctantly take on tasks passed down by others (2.1), reinforcing their desire for reciprocal and respectful collaboration, as participant 4 expressed:

Have some mercy on the GP! Sometimes they're just thinking: I have no idea either, off you go!

This underscores the central importance of mutual trust, an essential foundation for effective collaboration, whether at the centralized or local level. Early involvement is key to safeguarding professional autonomy and feasibility, but personal relationships are especially important at the local level, as participant 9 described:

Well, I’m truly blessed with a really great hospital, with medical specialists who approach general practitioners in a very respectful and friendly way, as equals. And we quickly developed a kind of personal bond. On top of that, we’d also see each other outside of work; at sports clubs, playing tennis or hockey and so on.

When GPs do deviate from guidelines or local agreements, it is usually after consulting with a specialist or based on a trusted relationship where challenges have been addressed together (11.2, 12.3).

Furthermore, centralization was frequently linked to effective collaboration, tied to organizational capacity, trust, reimbursement, and scale. Innovations are generally more successful when implemented through regional organizations, as they manage insurer funding and are trusted by GPs to coordinate care and innovation (13.1).

This trust is reciprocal; GPs noted that innovations are more effective when insurers allow them to respect their autonomy.

Perceptions of change varied depending on the GP’s additional engagements. Those involved nationally focused more on evidence standards, whereas those at local and regional levels, prioritized feasibility. Effective implementation thus requires tailoring strategies to the specific context (8.1). An elegant facilitator for achieving this was suggested by participant 15, who underlined that the most effective way is to engage GPs who convince other GPs: “So if you want someone to take action or implement a care pathway, you also need to speak their language.”

### Dynamic Theme 3: Continuity of Organizational Context

But the question is: OK, we have reimbursement now, we will have reimbursement in 2 years' time. But if I set something up, I want it to be sustainable. I don't want to say in 3 years' time: well, this is a very good project, but there is no money for it now. (Participant 10)

Alongside identity-related and social context, practical considerations also shape GPs’ willingness to adopt innovations, with continuity emerging as a central concern.

Beyond immediate reimbursement, GPs assess whether funding will be sustained long-term. Without this assurance, they are reluctant to invest in new projects or infrastructure.

Even modest reimbursement is a motivator (12.4). As participant 12 explained:

Well that you are appreciated, I think, and that your time is compensated. And even if you are employed, then yes, you just have to do the work and you still get exactly the same amount every month. But still, you do want to see your efforts… reflected, right?

This underscores the earlier mentioned desire for recognition of GPs, which need not be monetary, it may also come as time or staff support.

Innovation feasibility is closely tied to workload reduction, which GPs often cite as essential for successful implementation (9.2). Innovations must address problems GPs actually face, yet many noted a disconnect between their barriers and those perceived by specialists and policymakers:

Sometimes something seems, I think, very simple from the perspective of the hospital, while when you know your patients relatively well, you just know that sometimes a small thing can become more complicated. (Participant 11)

The growing number of NITs increases the need for consistent direction, as GPs tend to resist rapidly shifting policies and strongly value continuity (7.1).

New test strategies must also be simple, as complex procedures do not align with GP workflows. In MASLD, this can be a facilitator, as one GP with NIT experience noted the tests are easy to perform in practice. “Look, of course it’s not a huge effort… Every now and then an elevated ALT comes up, and then next time I’ll add a FIB4.” (Participant 11).

Beyond simplicity, practical accessibility for both patients and GPs also facilitates adoption (12.5). Ideally, tools should be integrated into existing workflows. In the Dutch setting, the structured cardiometabolic care system can serve as a facilitator for implementation of MASLD care pathways, as GPs noted these should be integrated into existing workflows. This is especially important for retesting at-risk patients after several years, as recommended in guidelines. Without such structure, GPs lack the capacity for this proactive recall.

This need for simplicity and integration reflects the broader reality of general practice, where GPs operate in small-scale settings and manage workflows independently. Feasibility is therefore highly individual and sensitive to external factors. This aligns with their pragmatic and holistic approach, as described by participant 10: “I think that if I were to follow the guidelines to the letter, I’d constantly be in conflict with my patients. Yes, I truly believe that, and it’s not because I don’t follow the guidelines, but because you’re providing personalized care and working within a specific context. And that simply can’t be captured in the guidelines.”

The participants accentuated that acknowledging this pragmatism in policy and guideline development may serve as a facilitator in the implementation process. Effective innovations should both structure care and accommodate the pragmatic decision-making that defines GP practice (14.3).

## Discussion

This qualitative study explored the barriers and facilitators to implementing noninvasive care pathways for MASLD in primary care and provides a deeper understanding of how GPs perceive and might adopt MASLD-related innovations. This study elucidates MASLD-specific implementation dynamics but also offers a conceptual and practical blueprint for similar innovations targeting chronic, underdiagnosed conditions in primary care. GPs showed strong willingness for participation in change, driven by commitment to patient well-being and intrinsic motivation for lifelong learning. However, this is continuously weighed against identity-related, social, and practical factors. Architects of care innovations must recognize that implementation in primary care is not merely logistical and guideline-based but a complex process involving cognitive, attitudinal, motivational, and relational dimensions. Ideally, effective implementation addresses all of these dimensions and is preceded by thorough mapping of local contexts. Without recognizing this complexity, innovation efforts risk limited uptake and sustainability. Implementation strategies should therefore be both evidence-based and context-sensitive.

Previous studies, both quantitative and qualitative, primarily frame barriers as knowledge deficits or organizational issues like reimbursement, implying that education and funding can effectively address these gaps.^9^, ^10^, ^11^^,^^14^^,^^28^^,^^29^ However, our findings indicate that barriers run deeper, rooted in social and cultural factors, including divergent views on quality care, professional identity, and relational dynamics between primary and specialist care. As Wensing and Grol argue, implementation science must address the attitudinal, motivational, and organizational factors shaping clinician behavior, to better reflect health care’s real-world complexity.^30^ A German study on chronic kidney disease guideline adaptation similarly highlighted the social and practical complexity of decision-making.^7^ Notably, our participants did not mention knowledge gaps as past barriers but stressed the importance of making learning relevant, viewing education as a prerequisite, not a barrier for implementation.

This aligns with the findings of the LOCATE intervention, an United Kingdom–based liver disease initiative, where GPs similarly did not view knowledge gaps as barriers, instead emphasizing contextual fit.^31^ The focus on knowledge in previous studies may partly reflect participant sampling of those with specific expertise or interest in MASLD, potentially leading to more supportive stances and less critical reflection. It may also stem from a reliance on conceptual or opinion-based input, whereas our interviews were grounded in day-to-day practice, though this distinction cannot be confirmed. The focus on knowledge gaps may further reflect the persistence of the information deficit model in scientific thinking, assuming that inaccurate beliefs stem from lack of knowledge and can be corrected with more or better information.^32^ Despite substantial evidence for the contrary, this model has persisted due to its intuitive appeal.^33^

A key implication for practice is the clear divergence in context, perspective, and evidence expectations between primary and specialist care. The hepatologist should recognize that GPs frequently disagree with specialists on the required level of evidence and stress the need for research within primary care settings, engaging GPs in generating and contextualizing evidence. While primary care’s role in MASLD management is increasingly recognized, the specific conditions motivating GPs to adopt MASLD-related innovations remain underexplored. This divergence reflects a deeper contrast in clinical reasoning: specialist guidelines often rely on frequentist logic derived from high-prevalence populations, whereas GPs apply Bayesian reasoning, interpreting evidence through prior experience, local prevalence, and individual context. This explains GPs’ demand for contextual relevance and their critical stance toward specialist-driven evidence. Ultimately, simply providing information is unlikely to change practice. Bridging the gap between primary and specialist care requires addressing the contextual and cognitive factors that shape how GPs evaluate evidence for each individual patient rather than taking specialist priorities as starting point.

Targeted research into GP-defined implementation prerequisites is urgently needed. In parallel, robust scientific implementation frameworks, such as organizational readiness for change^34^ and the Non-adoption, Abandonment, Scale-up, Spread, and Sustainability framework used by Vali et al for NIT implementation for MASLD, should guide interdisciplinary guideline development at both local and central levels.^35^ Our study highlights the importance of involving GPs, including those with limited MASLD experience or a critical stance, in future research efforts. Adaptation is more likely to succeed when diverse perspectives, including critical voices, are included.^36^

As discussed, GPs assess innovations based on their quality and impact on both overall health care and individual patients, particularly in relation to clinical guidelines, and they exercise substantial autonomy in deciding which innovations to adopt. Given their broad clinical scope, new initiatives must compete with other priorities and demonstrate sufficient benefit relative to time and resource investment. In the Dutch context, the GPs' strong reliance on national primary care guidelines issued by the Dutch College of General Practitioners (*Nederlands Huisartsen Genootschap*) may delay uptake of new evidence, hindering MASLD pathway implementation. However, the combination of independent practice and regional GP organizations offers opportunities to implement changes in parallel with guideline revisions. While these contextual features may not be universally transferable to other countries, considering autonomy, guideline reliance, and local organizational structures is essential for effectively advancing MASLD care and can inform tailored approaches in other health-care systems. Furthermore, although not explicitly brought forward by the present study’s participants, the presence and active engagement of patient advocacy groups for chronic liver disease may have the potential to positively impact the uptake of MASLD-related innovations.

Although not specific to MASLD, the theme of trustworthy partnership, reflecting the social dynamics of interdisciplinary collaboration, is highly relevant for implementation. GPs, like most people, often rely on intuition or professional instinct when evaluating change, an aspect that should not be overlooked in designing effective implementation strategies.

This study has several strengths. It offers novel insights into barriers and facilitators for integrating preventive hepatology for SLD in primary care, contributing meaningful insights to the field. By focusing on experiential knowledge and combining MASLD-specific and prior innovation experiences, we captured rich, in-depth perspectives on how GPs engage with care innovation and translate these perspectives into a conceptual blueprint that hepatologists can apply themselves.

Our sampling strategy ensured diverse representation across practice types, locations, and roles, enhancing the contextual richness and credibility. Rigorous analysis, following Braun and Clarke’s 6-phase framework,^20^ alongside iterative data collection and analysis, supported by regular discussions, strengthened analytic depth and dependability. Co-interviewing enabled more dynamic probing and deeper exploration of participant perspectives, enhancing richness of the data.

Our interdisciplinary team, spanning hepatology, internal medicine, general practice, and qualitative research, enhanced understanding and yielded nuanced interpretation, especially concerning Bayesian reasoning and interdisciplinary collaboration.

Finally, our thematic analysis balanced semantic clarity with contextual variation, capturing the complexity and interconnected nature of implementation processes.

Several limitations also need consideration. Limited MASLD-specific experiential knowledge meant most reflections were analogies to other innovations. While this provided valuable insights into implementation dynamic complexity, some interpretations were not grounded in direct experience with MASLD-specific innovations.

As interview-based data, our findings capture perceptions rather than practice, and omit certain practical factors, better addressed through methods like participatory action research.^37^

As qualitative research is not intended to produce generalizable findings, this study aimed for transferability, providing insights into complex contexts to inform understanding and generate hypotheses.

This is particularly relevant given the distinct Dutch context, where GPs act as gatekeepers to specialist care and face unique reimbursement structures. Nonetheless, many social themes and the application of Bayesian reasoning under relative uncertainty are broadly applicable and can enrich understanding diverse health-care systems. Providing detailed contextual information enables readers to applicate our findings to their own setting.

Interviewers N.B. and S.D., both PhD candidates researching noninvasive testing for MASLD, brought a distinct perspective to this GP-focused study, which may have influenced data collection and interpretation. To mitigate this, the multidisciplinary team included GPs in both the study’s conceptualization and analysis. Reflexive dialogue and regular team discussions helped identify potential biases and explore differing interpretations.

Although dual interviewing can increase power imbalance in qualitative research, N.B. and S.D., as junior researchers interviewing experienced GPs, did not experience this dynamic.

## Conclusion

This study challenges current perspectives on implementing MASLD-related innovations by showing that innovation in primary care is a complex process that extends beyond financial incentives or educational interventions. Although GPs demonstrate strong willingness to adopt new care pathways, there is a constant trade-off with identity-related, relational, and practical factors. Clinical decision-making in primary care follows a distinct logic, characterized by lower disease prevalence, diagnostic uncertainty, and a focus on tangible patient impact.

In MASLD, complexity is further heightened by limited experiential knowledge, low perceived urgency, and skepticism toward the current evidence base. GPs assess innovations pragmatically through a Bayesian lens, balancing expected benefit against disruption to continuity, time investment, and workload, while placing high value on trustworthy partnerships. Education remains pivotal, but should be introduced just-in-time.

Implementation strategies that acknowledge this reasoning, by underscoring contextual fit, practical feasibility, and collaboration, are more likely to succeed. Sustainable implementation requires moving beyond awareness and reimbursement toward codesigned, workflow-integrated, and practice-based approaches that resonate with GPs’ clinical reasoning, professional identity, and local context.

## Supplementary Materials

Supplementary Materials 1–4

Extended PDF
