# Supplementary material for: Beyond Awareness: A Qualitative Assessment of Barriers and Facilitators to Implementing Metabolic Dysfunction–Associated Steatotic Liver Disease Care Pathways in Primary Care
Source: Gastro Hep Adv. 2026 May 5;5(8):100991. doi: 10.1016/j.gastha.2026.100991 (PMC13262144; doi:10.1016/j.gastha.2026.100991)
Supplement: Supplementary Materials 1–4 [file mmc1.pdf]

Supplemental Material

Beyond awareness: a qualitative assessment of barriers and facilitators to implementing MASLD care pathways in primary care

Shared first authors: Stan Driessen, Leonard (Niels) D. Broekman

Table of contents:

Supplemental material 1. Description of Dutch healthcare system	P. 2
Supplemental material 2. Final interview guide, translated to English	P. 3
Supplemental material 3. Final interview guide, original Dutch version	P. 6
Supplemental material 4. Complete description of how analytic sufficiency was reached	P. 9

Supplemental material 1. Description of Dutch healthcare system

The Dutch healthcare system can be defined as a system of regulated market competition consisting of three distinct markets.(1) First, the health insurance market between patient and insurer, second, the health care purchasing market between insurer and provider, and third, the health care provision market between provider and patient. All Dutch inhabitants are obliged to arrange a basic insurance plan with one of the health insurance companies on the insurer's market. All health insurers are legally obliged to accept any applicant for their basic insurance package, ensuring universal access regardless of health status. Next to the individual premiums for mandatory basic health insurance, the Dutch healthcare system is primarily funded through employer and government contributions. Basic insurance plans cover all consultations in primary care and keep out-of-pocket costs low. GP compensation is a mix of capitation and fee-for-service payments. In the Dutch system, GPs are the first point of contact for most medical concerns and management of several chronic conditions e.g. type 2 diabetes mellitus and COPD as well as cardiovascular risk management. Furthermore, GPs function as gatekeepers providing personalised referrals to emergency departments or outpatient clinics in regional hospitals, university hospitals, or independent treatment centres. For most outpatient, specialist care, patients pay a maximum out-of-pocket deductible per year. Health insurers contract with hospitals and independent treatment centres, and medical specialists are generally organised within partnerships who are on their turn contracted by the hospitals. Hospitals are predominantly private, independently managed non-profit entities who operate under national regulations. Within this system, and through freedom of choice for patients, hospitals are expected to compete on quality and efficiency, whereas primary care is exempted from these incentives. The Dutch government has a regulatory role, overseeing coverage decisions and managing risk equalisation measures that compensate insurers for taking on high-risk patients.

Supplemental material 2. Final interview guide, translated to English

**BARRIERS AND FACILITATORS IN IMPLEMENTING CARE PATHWAYS FOR MASLD IN DUTCH PRIMARY CARE**		
**Background Information**	Interviewers	S. Driessen<br>L.D. Broekman
	Supervisors	A. de la Croix<br>M.C. Mak-van der Vossen<br>M.E. Tushuizen<br>O.R. Maarsingh<br>A.G. Holleboom
	Organisation	Vascular Medicine & General pratice/Family medicine, Amsterdam UMC
**Introduction**	• First of all, I want to thank you for taking the time to participate in this interview.<br>• Do you prefer to be addressed formally or informally?<br>• I'd like to give you some background about this research project, and then explain how I plan to conduct this interview.<br>• If you have any questions before the interview starts, you can ask them after the explanation.	
**Background**	• Currently, there is global interest in implementing care pathways for diagnosing liver fibrosis due to metabolic dysfunction-associated steatotic liver disease (MASLD), previously known as non-alcoholic fatty liver disease (NAFLD). In the Netherlands, there is no standardised testing for liver fibrosis within a care pathway.<br>• A Dutch multicenter study is currently underway investigating the effectiveness of these pathways across different levels of care. However, it is equally important to assess whether these pathways are also desirable and feasible in daily practice.	
**Research aim**	• To explore the general practitioner's perspective on perceived barriers and facilitators for implementing MASLD fibrosis care pathways.	
**Privacy**	• The data will be used for publication in a scientific article.<br>• Your personal information will only be accessed by our research team at Amsterdam UMC.<br>• In the article, personal data will be pseudonymised. The results will not be traceable back to you.	
**Duration**	• This interview will take approximately 30 to 40 minutes.	
**Additional questions**	• Do you have any additional questions?	

**Recording**	• This interview will be audio recorded<br>• Legally, I am required to ask for your permission to do so.<br>• START RECORDING<br>• Do I have your permission to record this interview?
**INTRODUCTORY QUESTION**	
**Interviewee Background**	• Can you briefly tell me who you are, where you work, and what kind of general practice you work in?<br>• How many years have you been working as a GP?<br>• Do you have a specific area of expertise?
**Domain 1:** *Experiences with MASLD in Daily Practice*	Can you describe how you approach MASLD in your daily practice?<br><br>• Have you ever referred a patient for hepatic steatosis?<br>• Do you currently have a patient with MASH-cirrhosis?
**Domain 2:** *Other Innovations in General Practice*	What are your experiences with previous innovations in primary care? What facilitated them, and what made them difficult?<br><br>• **Can you give an example?**<br>• Can you draw a parallel with hepatic steatosis?<br>• What has your experience been with communication from the hospital regarding this care (or previous implementations)?<br>• What aspects are often overlooked?<br>• What makes GPs enthusiastic about certain changes?
**CLOSING QUESTION**	
**Summary**	We are nearing the end of this interview. As a final question, could you briefly summarise:<br>• The most important facilitators for implementing these non-invasive MASLD tests in your practice, from your perspective?<br>• The most important barriers to implementing these non-invasive MASLD tests in your practice, from your perspective?
**Final Remark**	• Would you like to add anything that we haven't discussed yet?
**CLOSING THE INTERVIEW**	
**Conclusion**	• That brings us to the end of this interview.
**Thanks**	• I sincerely want to thank you for your time and participation. I really enjoyed this conversation and I hope you did too.
**Next Steps**	• This interview will be transcribed verbatim.

	• Afterwards, I will send you a one-page summary for review. This gives you the opportunity to add anything that may have been missed.<br>• The main findings from this interview will be compared with the main findings from other interviews.<br>• After analysis, the results will be incorporated into a scientific article.<br>• Would you like to receive a message when the paper is published?<br>• Do you have any further questions?<br>• STOP RECORDING

Supplemental material 3. Final interview guide, original Dutch version

<table>
<tr><th colspan="3">VALKUILEN EN BEVORDERAARS IN HET IMPLEMENTEREN VAN ZORGPADEN VOOR MASLD IN DE NEDERLANDSE EERSTE LIJN ZORG</th></tr>
<tr><td rowspan="3">Achtergrond informatie</td><td>Interviewers</td><td>S. Driessen
L.D. Broekman</td></tr>
<tr><td>Supervisors</td><td>A. de la Croix
M.C. Mak-van der Vossen
M.E. Tushuizen
O.R. Maarsingh
A.G. Holleboom</td></tr>
<tr><td>Organisatie</td><td>Vasculaire geneeskunde & Huisartsgeneeskunde, Amsterdam UMC</td></tr>
<tr><td>Introductie</td><td colspan="2">• Eerst en vooral wil ik u bedanken voor vrijmaken van uw tijd om deel te nemen aan dit interview.
• Heeft u liever dat ik vousvoyeer of tutoyeer?
• Ik wil u wat achtergrondinformatie geven over dit onderzoeksproject. Daarna zal ik uitleg geven over de manier waarop ik dit interview wil afnemen.
• Als u vragen hebt voordat het interview begint, kunt u ze na de uitleg stellen.</td></tr>
<tr><td>Achtergrond</td><td colspan="2">• Momenteel is er wereldwijd veel belangstelling voor het implementeren van zorgpaden voor diagnostiek naar leverfibrose als gevolg van metabolic dysfunction associated steatotic liver disease (MASLD), voorheen non-alcoholic fatty liver disease (NAFLD). In Nederland wordt momenteel nog niet standaard getest op leverfibrose in een zorgpad.
• Er loopt momenteel een Nederlandse multicenter studie die de effectiviteit van deze zorgpaden onderzoekt in meerdere lijnen van zorg. Echter is het ook van belang om te weten of de zorgpaden ook gewenst zijn en uitvoerbaar zijn in de praktijk.</td></tr>
<tr><td>Doel onderzoek</td><td colspan="2">• In kaart brengen van perspectief van de huisarts op gepercipieerde barrières en facilitatoren voor zorgpadimplementatie voor MASLD-fibrose.</td></tr>
<tr><td>Privacy</td><td colspan="2">• De gegevens worden gebruikt voor publicatie in een artikel.
• Uw persoonlijke gegevens worden alleen ingezien door ons onderzoeksteam in het Amsterdam UMC.
• In het artikel worden persoonlijke gegevens gepseudonimiseerd. De resultaten van dit onderzoek zullen dus niet tot u herleidbaar zijn.</td></tr>
</table>

**Duur**	• Dit interview duurt ongeveer 30 tot 40 minuten.
**Aanvullende vragen**	• Heeft u nog aanvullende vragen?
**Opname**	• Van dit interview wordt een geluidsopname gemaakt<br>• Het is wettelijk verplicht dat ik toestemming vraag om dit te doen.<br>• START OPNAME<br>• Heb ik uw toestemming om dit interview op te nemen?
**INLEIDENDE VRAAG**	
**Achtergrond geïnterviewde**	• Kunt u mij in het kort vertellen wie u bent, waar u werkt en in wat voor soort huisartspraktijk u werkt?<br>• Hoeveel jaar werkt u al als huisarts?<br>• Heeft u een specifiek expertisegebied?
**Domein 1:**<br>*Ervaringen met MASLD in de dagelijkse praktijk*	Kunt u vertellen hoe u omgaat met MASLD in uw dagelijkse praktijk?<br><br>• Heeft u wel eens een patiënt verwezen voor leversteatose?<br>• Heeft u een patiënt in de praktijk met MASH-cirrose?
**Domein 2:**<br>*Andere innovaties*	Wat zijn uw ervaringen met eerdere innovaties in de huisartsenzorg? Wat heeft dit gefaciliteerd, wat maakte het lastig?<br><br>• **Kunt u daar een voorbeeld van geven?**<br>• Kunt u een parallel trekken met leversteatose?<br>• Hoe is de ervaring met communicatie vanuit het ziekenhuis omtrent deze zorg (of eerdere implementaties)?<br>• Welke zaken worden meestal over het hoofd gezien?<br>• Wat maakt dat huisartsen ergens enthousiast voor worden?
**AFSLUITENDE VRAAG**	
**Samenvatting**	We naderen het einde van dit interview. Als laatste vraag: kunt u kort samenvatten:<br>• De belangrijkste facilitators voor de implementatie van deze niet-invasieve MASLD-testen in uw praktijk vanuit uw perspectief?<br>• De belangrijkste barrières voor de implementatie van deze niet-invasieve MASLD-testen in uw praktijk vanuit uw perspectief?
**Slotopmerking**	• Wilt u nog iets toevoegen dat we nog niet hebben besproken?
**AFSLUITING INTERVIEW**	
**Afsluiting**	• Daarmee is dit interview tot het einde gekomen

**Dank**	• Ik wil u oprecht bedanken voor uw tijd en deelname aan dit interview. Ik vond het erg leuk om dit gesprek met u te hebben en ik hoop dat u het ook zo hebt ervaren.
**Vervolgstappen**	• Dit interview zal woordelijk worden getranscribeerd.<br>• Daarna stuur ik een samenvatting van ongeveer één pagina ter controle. Dit geeft u de mogelijkheid om iets toe te voegen aan wat er is besproken.<br>• De belangrijkste resultaten van dit interview worden vergeleken met de belangrijkste resultaten van de andere interviews.<br>• Na analyse zullen de resultaten verwerkt worden in een nog te publiceren artikel.<br>• Wilt u een bericht ontvangen als er een paper gepubliceerd gaat worden?<br>• Heeft u verder nog vragen?<br>• STOP OPNAME

Supplemental material 4. Complete description of how analytic sufficiency was reached

We determined the end of data collection based on the methodologically grounded principle of analytic sufficiency, which offers a more nuanced and robust alternative to the increasingly contested concept of data saturation. Following the iterative nature of thematic analysis, data collection and analysis were conducted concurrently. As interviews progressed, we observed decreasing novelty in the coding process, and the core themes became increasingly well-defined and richly supported.

We applied the concept of information power, considering the study aim, sample specificity, quality of dialogue, and the strength of the established themes. While our sample was diverse in terms of practice type, MASLD experience, and involvement in innovation, the relatively narrow study aim, in combination with the richness of the data due to co-interviewing, led to high information power.

Analytic sufficiency was confirmed when no new relevant dimensions emerged and existing themes were internally coherent. The four themes were fully disentangled from the data after interview 11. The remaining four interviews mostly added depth to the themes and their interrelations. The broad context of theme “Trustworthy partnership between specialist care and primary care” and theme “Continuity of organisational context”, indicate that it would have been possible to possibly gain more in-depth insights with more interviews. Given the MASLD-specific research question, we decided after team discussions that this would not have added additional value to this specific context and we concluded that additional interviews would likely lead to redundancy rather than added insight. This decision was reached collaboratively by the entire research team after 15 interviews. Member checking was not conducted, as participants’ accounts were concrete and closely linked to their professional experiences, leaving little scope for reinterpretation. While member checking is well-established in qualitative research, it is not universally necessary or desirable, especially in contexts like qualitative health studies where clear, descriptive data already robustly support thematic findings.(2)

References

1. Misser NS, Versendaal PhD J, Methorst M, Stork B. Dutch Healthcare: An Overview and Application. 2014.
2. Thomas DR. Feedback from research participants: are member checks useful in qualitative research? Qualitative Research in Psychology 2017;14:23-41.

COREQ (COnsolidated criteria for REporting Qualitative research) Checklist

A checklist of items that should be included in reports of qualitative research. You must report the page number in your manuscript where you consider each of the items listed in this checklist. If you have not included this information, either revise your manuscript accordingly before submitting or note N/A.

Topic	Item No.	Guide Questions/Description	Reported on Page No.
**Domain 1: Research team and reflexivity**			
Personal characteristics			
Interviewer/facilitator	1	Which author/s conducted the interview or focus group?	11
Credentials	2	What were the researcher's credentials? E.g. PhD, MD	9
Occupation	3	What was their occupation at the time of the study?	9
Gender	4	Was the researcher male or female?	x
Experience and training	5	What experience or training did the researcher have?	9
Relationship with participants			
Relationship established	6	Was a relationship established prior to study commencement?	10
Participant knowledge of the interviewer	7	What did the participants know about the researcher? e.g. personal goals, reasons for doing the research	supplemental [illegible]
Interviewer characteristics	8	What characteristics were reported about the inter viewer/facilitator? e.g. Bias, assumptions, reasons and interests in the research topic	9, 11, 25
**Domain 2: Study design**			
Theoretical framework			
Methodological orientation and Theory	9	What methodological orientation was stated to underpin the study? e.g. grounded theory, discourse analysis, ethnography, phenomenology, content analysis	9, 11
Participant selection			
Sampling	10	How were participants selected? e.g. purposive, convenience, consecutive, snowball	10
Method of approach	11	How were participants approached? e.g. face-to-face, telephone, mail, email	10
Sample size	12	How many participants were in the study?	11
Non-participation	13	How many people refused to participate or dropped out? Reasons?	10, 11
Setting			
Setting of data collection	14	Where was the data collected? e.g. home, clinic, workplace	10
Presence of non-participants	15	Was anyone else present besides the participants and researchers?	11
Description of sample	16	What are the important characteristics of the sample? e.g. demographic data, date	13
Data collection			
Interview guide	17	Were questions, prompts, guides provided by the authors? Was it pilot tested?	10, supplemen
Repeat interviews	18	Were repeat inter views carried out? If yes, how many?	11, supplemer
Audio/visual recording	19	Did the research use audio or visual recording to collect the data?	11
Field notes	20	Were field notes made during and/or after the inter view or focus group?	11
Duration	21	What was the duration of the inter views or focus group?	11
Data saturation	22	Was data saturation discussed?	11, supplemen
Transcripts returned	23	Were transcripts returned to participants for comment and/or	supplemental [illegible]

Topic	Item No.	Guide Questions/Description	Reported on Page No.
		correction?	
**Domain 3: analysis and findings**			
Data analysis			
Number of data coders	24	How many data coders coded the data?	11,12
Description of the coding tree	25	Did authors provide a description of the coding tree?	11
Derivation of themes	26	Were themes identified in advance or derived from the data?	9, 11
Software	27	What software, if applicable, was used to manage the data?	11
Participant checking	28	Did participants provide feedback on the findings?	supplemental
Reporting			
Quotations presented	29	Were participant quotations presented to illustrate the themes/findings? Was each quotation identified? e.g. participant number	13-20
Data and findings consistent	30	Was there consistency between the data presented and the findings?	13-20, 21-23
Clarity of major themes	31	Were major themes clearly presented in the findings?	13
Clarity of minor themes	32	Is there a description of diverse cases or discussion of minor themes?	13-20

Developed from: Tong A, Sainsbury P, Craig J. Consolidated criteria for reporting qualitative research (COREQ): a 32-item checklist for interviews and focus groups. *International Journal for Quality in Health Care*. 2007. Volume 19, Number 6: pp. 349 – 357

Once you have completed this checklist, please save a copy and upload it as part of your submission. DO NOT include this checklist as part of the main manuscript document. It must be uploaded as a separate file.
