# Supplementary material for: Beyond Awareness: A Qualitative Assessment of Barriers and Facilitators to Implementing Metabolic Dysfunction–Associated Steatotic Liver Disease Care Pathways in Primary Care
Source: Gastro Hep Adv. 2026 May 5;5(8):100991. doi: 10.1016/j.gastha.2026.100991 (PMC13262144; doi:10.1016/j.gastha.2026.100991)
Supplement: Extended PDF [file mmc2.pdf]

Gastro Hep Advances 2026;5:100991

ORIGINAL RESEARCH—CLINICAL

Beyond Awareness: A Qualitative Assessment of Barriers and Facilitators to Implementing Metabolic Dysfunction–Associated Steatotic Liver Disease Care Pathways in Primary Care

Stan Driessen,[1,2,*] Leonard (Niels) D. Broekman,[1,2,*] Anne de la Croix,[3] Marianne C. Mak-Van der Vossen,[3] Maarten E. Tushuizen,[4] Otto R. Maarsingh,[3] and Adriaan (Onno) G. Holleboom[1,2]

[1]*Department of Vascular Medicine, Amsterdam UMC location University of Amsterdam, Amsterdam, the Netherlands;* [2]*Department of Gastroenterology and Hepatology, Amsterdam UMC location University of Amsterdam, Amsterdam, the Netherlands;* [3]*Department of General Practice/Family Medicine, Amsterdam UMC location University of Amsterdam, Amsterdam, the Netherlands; and* [4]*Department of Gastroenterology and Hepatology, LUMC, Leiden University, Leiden, the Netherlands*

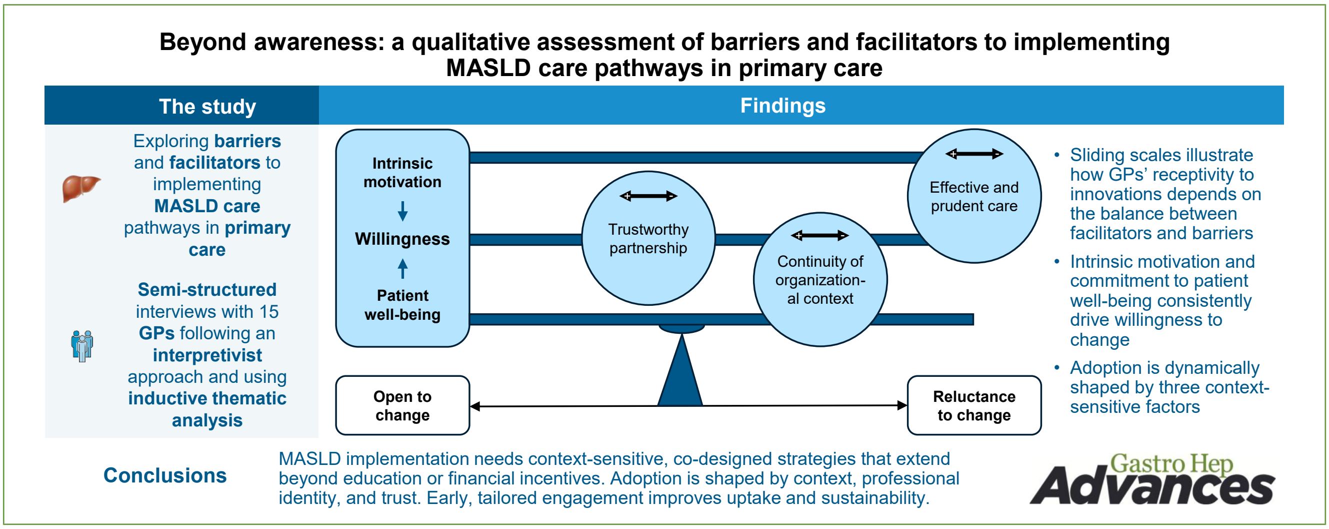

BACKGROUND AND AIMS: Health-care systems must adapt to a rising burden of metabolic dysfunction–associated steatotic liver disease (MASLD), yet implementation in primary care remains challenging. While existing strategies often target knowledge deficits, our study uniquely examines how cognitive, contextual, and experiential factors shape general practitioners' (GPs) decision-making. This qualitative study explores GPs' experiences with MASLD care and previous care innovations to identify barriers and facilitators to implementing MASLD care pathways in primary care. **METHODS:** We conducted semistructured interviews with 15 Dutch GPs from diverse backgrounds, enhancing data richness through cointerviewing. Guided by an interpretivist approach, we applied inductive thematic analysis per Braun and Clarke. Maximum variation sampling was applied, and interviewing continued until sufficient information power was achieved for analytic sufficiency. **RESULTS:** Findings were organized using a sliding scale framework: 1 consistent theme promoting innovation and 3 dynamic themes that can facilitate or impair innovation. GPs expressed strong intrinsic motivation for innovations, rooted in patient-centeredness and lifelong learning, yet continuously weighed this against identity-related, relational, and practical considerations. GPs stressed that primary care is not merely hospital care at a different place. They evaluate innovations pragmatically through a Bayesian lens, considering prior disease probability, test performance, and feasibility, combined with assessment of individual patient benefit. Key barriers included low perceived urgency for MASLD, driven by limited experiential knowledge

*Denotes co-first authorship.

Abbreviations used in this paper: **GP, general practitioner; MASLD, metabolic dysfunction–associated steatotic liver disease; NIT, noninvasive test; SLD, steatotic liver disease.**

Most current article

© 2026 The Author(s). Published by Elsevier Inc. on behalf of American Gastroenterological Association Institute. This is an open access article under the CC BY license (http://creativecommons.org/licenses/by/4.0/).

2772-5723
https://doi.org/10.1016/j.gastha.2026.100991

and skepticism toward specialist-derived evidence. GPs perceived trust-based partnerships, organizational continuity, and contextual fit as critical. **CONCLUSION:** Implementing hepatology-derived innovations in primary care is complex and extends beyond financial incentives or educational interventions. Effective strategies require codesigned, context-sensitive, and practice-integrated approaches that align with GPs' distinct logic and perspectives, including of those less engaged with MASLD.

Keywords: Metabolic Dysfunction–Associated Steatotic Liver Disease; Interview; General Practitioner; Care Innovation; Care Pathway

Introduction

As disease burden rises, adaptation of the health-care system is often necessary to optimize health care. A central component is changing physician behavior, which remains a widely recognized implementation challenge.[1] The rising prevalence and severity of the steatotic liver diseases (SLDs), largely driven by the increasing incidence of metabolic dysfunction–associated steatotic liver disease (MASLD) and metabolic dysfunction–associated alcohol-related liver disease, bring an accompanying care organization conundrum, exemplifying these concepts. The current worldwide prevalence of MASLD is estimated at 31% of the adult population with substantial regional differences and is expected to increase over the coming years. Substantial regional variation in MASLD prevalence exists, ranging between 25% in Western Europe and 44% in Latin America, respectively.[2] Metabolic dysfunction–associated steatohepatitis drives the advanced stages of fibrosis and cirrhosis, leading to substantial and gradually increasing health-care costs.[3] Multiple national and international guidelines recommend noninvasive tests (NITs) for hepatic fibrosis as the current best practice for diagnosing and managing SLD.[4,5] However, substantial discrepancies between SLD reference guidelines and real-world practice have been observed.[6] This challenge extends beyond SLD, as adherence to established secondary prevention programs, such as those for diabetic retinopathy and chronic kidney disease, remains complex, particularly in primary care.[7,8]

In the case of MASLD, disconnections between guidelines and practice are often attributed to limited provider awareness and knowledge, prompting a predominant focus on education to improve adherence.[9–11] However, broader change management frameworks highlight cognitive, attitudinal, and contextual domains that remain underexplored in SLD literature yet might be crucial in sustainable implementation of care pathways.[12] Earlier qualitative studies have emphasized general perceptions of NITs and MASLD care, while overlooking experiential and tacit knowledge: the lived, practice-based insights crucial to understanding complex care dynamics.[13] In MASLD, this poses a particular challenge, as limited awareness of the disease burden restricts opportunities to develop experiential understanding.[14] Yet, capturing the perspectives of a broad range of clinicians, including those with limited MASLD experience, is essential to explain suboptimal uptake of MASLD innovations. Experiential insight is often lacking, especially in primary care, where advanced MASLD is less prevalent than in specialized settings.[2]

From a Bayesian perspective, clinical decision-making involves updating disease probability based on prior prevalence and test performance. This framework is vital in illustrating the distinction between primary and specialized care, especially in diagnostic use and interpretation of test results.[15] In primary care, the lower prevalence of advanced MASLD necessitates a distinct approach, as general practitioners (GPs) must interpret inconclusive evidence within broader differentials and with different test performance. Paradoxically, despite encountering fewer advanced MASLD cases, GPs remain central to screening and prevention, as they manage the majority of at-risk patients through cardiometabolic care programs. Knowledge transfer from specialist to primary care is often gradual, shaped by differences in priorities, context, and perceived relevance. Current MASLD guidelines originated in specialist settings, but dedicated primary care guidance is still lacking, and GPs remain underrepresented in related research.[16,17] Recognizing this stepwise nature of knowledge transfer highlights the need for earlier, proactive primary care engagement in addressing emerging burdens like MASLD.

Although knowledge deficits have been identified as barriers to MASLD care pathway implementation, critical gaps remain in understanding how primary care professionals experience, interpret, and navigate recommendations in routine practice. This study explored GPs' experiences with MASLD and prior health-care innovations, in particular secondary prevention programs, to inform strategies for MASLD screening and management. In this qualitative study, we investigated barriers and facilitators to implementing MASLD fibrosis care pathways in Dutch primary care, a setting well suited given GPs' gatekeeping role[18] and established cardiometabolic care infrastructure.[19] While MASLD is the focal point of this study, the insights into GPs' reasoning, innovation uptake, and contextual navigation may also inform implementation of other secondary prevention pathways in primary care.

Methods

Study Design

We conducted a qualitative study using semistructured interviews, maintaining an interpretivist approach, which views knowledge as shaped by social interaction and open to multiple interpretations.[20] Consequently, we acknowledge that our perspectives and experiences influence data collection and interpretation. Our interdisciplinary team includes a hepatologist (M.T.), an endocrinologist/vascular medicine specialist with expertise in MASLD (O.H.), 2 GPs, 1 focused on health-

care continuity (O.M.), and 1 on qualitative research and professional identity in medical education (M.M.), a qualitative health and education researcher with a background in linguistics and ample experience in supervising qualitative research projects and teaching courses on qualitative research (A.C.), and 2 MD-PhD candidates (N.B. and S.D.) studying MASLD-fibrosis care pathways implementation, with interests in health care's socioeconomic context and interdisciplinary collaboration. The MD-PhD candidates were trained, coached, and supervised in qualitative research by 1 GP and the qualitative health and education researcher, and completed formal courses. Our diverse backgrounds fostered rich discussion and nuanced interpretation of the health-care context.

Study Setting

In the Dutch health-care system, GPs are the first point of contact for most medical concerns and function as gatekeepers, providing personalized referrals to specialized care.[18] Primary care practices are typically small and independent, closely collaborating with physiotherapists, pharmacists, and neighboring GPs, either informally or through regional primary care organizations. Cardiovascular risk management is often delivered by practice nurses under GP supervision. An extensive description of the Dutch health-care system is provided in Supplementary Material 1.

Sampling of Participants

We used maximum variation sampling to recruit GPs with diverse backgrounds, including those with and without roles in management, innovation, or guideline development; varying experience with NITs; and differing interests in type 2 diabetes, cardiovascular risk management, and lifestyle medicine.[21] We also ensured variation in practice type (solo, group, multidisciplinary), location (urban/rural), age, and gender to capture a broad range of perspectives. Participants were identified via snowball sampling and primary care organization websites, then contacted directly. Twenty-nine GPs were contacted in total, and interviews were scheduled within several weeks.

The study followed the Declaration of Helsinki, was approved by the Amsterdam UMC Ethics Committee and exempted from further review (2025.0321), followed institutional and General Data Protection Regulation guidelines, and obtained audio-recorded informed consent. Data were deidentified and handled per Amsterdam UMC Public Health research code.

Development of Interview Guide

We developed an interview guide to explore barriers and facilitators for implementing MASLD fibrosis care pathways in Dutch primary care, informed by literature on GPs' MASLD knowledge, referral patterns, and guideline-practice gaps.[6,14,22] The initial guide covered 4 domains: MASLD knowledge and vision, diagnostic methods, implementation, and health-care organization. After 3 pilot interviews (conducted by S.D.), the guide was refined to 2 domains: experiences and approaches to MASLD in daily practice and experiences with past health-care innovations and their relevance to MASLD. After observing limited MASLD-specific experiential knowledge in the pilots, we broadened the scope to include GPs' experiences with innovations for other diseases. Emphasis during interviews varied depending on GPs' MASLD experience, innovation background, and previous roles. The final interview guide is provided in Supplementary Material 2.

Data Collection

Between January 2024 and May 2025, we conducted 15 semistructured interviews with GPs, either in person or via video call, with all participants providing recorded consent. To become familiar with the interview guide, S.D. and N.B. each conducted 1 solo interview, followed by a joint interview to align styles. As cointerviewing improved probing and depth, it was continued with alternating leader roles.[23] Interviews lasted 34–55 minutes (median = 42), and field notes were written during each interview. All interviews were recorded and transcribed verbatim. Data interpretation was discussed in team meetings, and data collection was concluded based on sample diversity and analytic sufficiency.[24] We applied the concept of information power to assess when sufficient depth and variation had been achieved.[25] Details are provided in Supplementary Material 3.

Data Analysis

We conducted an inductive thematic analysis following Braun and Clarke's 6-phase framework.[20] Data analysis began after the first interview and proceeded iteratively alongside data collection. To ensure trustworthiness and rigor, an audit trail was kept, documenting decisions on codebook development, theme refinement, and reflexive discussions. S.D. coded all transcripts inductively. S.D. and N.B. selected 3 context-rich interviews that were independently coded by N.B., after which code trees were discussed and merged. Thereafter, N.B. coded 1 in every 3 interviews, with S.D. consulting N.B. on new codes or patterns.[26] Coding was done in MAXQDA 2022, and a native English speaker with Dutch fluency checked translations of selected quotes. Findings were organized around key semantic topics, capturing how participants conceptualized MASLD and previous care innovations. N.B. and S.D. regularly discussed theme development with each other and the broader team, focusing on rich and overlapping codes. The written analysis was cross-checked against codes and excerpts to ensure accuracy and representativeness, supporting a nuanced interpretation and avoiding overgeneralization. Final manuscript drafts were discussed by all authors to enhance confirmability and ensure alignment with the data.[27]

Results

Our findings are based on interviews with 15 GPs with varying backgrounds. Seven were women, 5 were involved in national GP societies/organizations, 3 in regional organizations, 5 in MASLD initiatives, and both urban and rural areas were represented (Table 1).

We structured our findings using a sliding scale framework illustrating the continuous balancing act that GPs face when considering innovation (Figure). We describe 1 consistent theme that universally promotes change, and 3 dynamic themes that can either facilitate or hinder change depending on context. We begin with the

Table 1. Descriptive Information About Participating General Practitioners

Characteristics	n (%, unless specified other)
Gender	
Female	7 (46.7%)
Male	8 (53.3%)
Age	
Median (range)	52 (36–65)
Practice type[a,b]	
Solo	4 (26.7%)
Duo	1 (6.7%)
Solo+	2 (13.3%)
Small group	2 (13.3%)
Medium-large group[f]	4 (26.7%)
Nonpracticing	2 (13.3%)
Patient population	
Median (range)	5100 (2000–13,000)
External activities next to regular clinical practice	
Involved in national society[c]	5 (33.3%)
Involved in regional society	3 (20.0%)
Involved in MASLD initiatives[d]	5 (33.3%)
Academic	3 (20.0%)
Area[e]	
Urban	8 (53.3%)
Suburban	5 (33.3%)
Rural	2 (13.3%)

[a]A distinction is made between GPs that practice together, under the same name, in a fully integrated group practice, and GPs that practice in a solo practice (here named "Solo+," or "HOED" in Dutch), but under the same roof with several other solo practices, thereby sharing equipment, space, staff, etc.
[b]Small group practices are defined as practices with up to 4 permanently practicing GPs, while medium-large group practices are defined as practices with 5 or more permanently practices GPs.
[c]Dutch College of General Practitioners (NHG) or National Association of General Practitioners (Landelijke Huisartsen Vereniging).
[d](Care pathway) studies or outreach endeavors.
[e]Urban, $\geq$1500 inhabitants/km^2; suburban, 500/km^2–1500/km^2; rural, $<$500/km^2.
[f]One of the GPs that practices in a group practice is practice owner as well.

consistent theme: The GPs' strong willingness for change, and explore its underlying drivers. The 3 dynamic themes are categorized according to identity-related, social, and practical dimensions, while acknowledging their complexity and interrelatedness. Supporting quotes are presented in-text or provided in Table 2 and referenced to using quote-specific denotations.

Overarching Consistent Theme: Willingness for Change

> *At the same time, I still believe that if it can be done in primary care, then it should be done in primary care… So, if you, as a patient, don't need to go to secondary care because your GP has safely ruled out that it's necessary, well that's amazing right? (Participant 8)*

As an overarching theme, GPs consistently express a strong willingness to engage in care transformation, including adopting new tasks. This motivation is largely driven by their commitment to patient well-being. As participant 4 stated:

> *Look: doctors, all doctors, want the best for the patient sitting in front of them.*

GPs also express an urgency for lifelong learning, fueling their interest in new health-care approaches (12.1). However, intrinsic motivation varies by personal preferences, interests, and propensity to adopt innovations (9.1).

GPs continuously balance this willingness to innovate with external constraints, resulting in a dynamic trade-off between openness and reluctance, as participant 12 noted: "Yes, because naturally you want to serve your patients well… So I am very willing to do that, but I do need the space and the opportunity to do so. Also, if for example, I can't hire extra staff because there simply isn't funding for it, then at some point it's obviously going to cause friction."

Dutch GP practices range mostly from small group practices to solo practices, with GPs retaining substantial autonomy and financial responsibility. This dual role, both health-care providers and entrepreneurs, results in careful cost-benefit considerations and underscores the importance of continuity (quote 14.1).

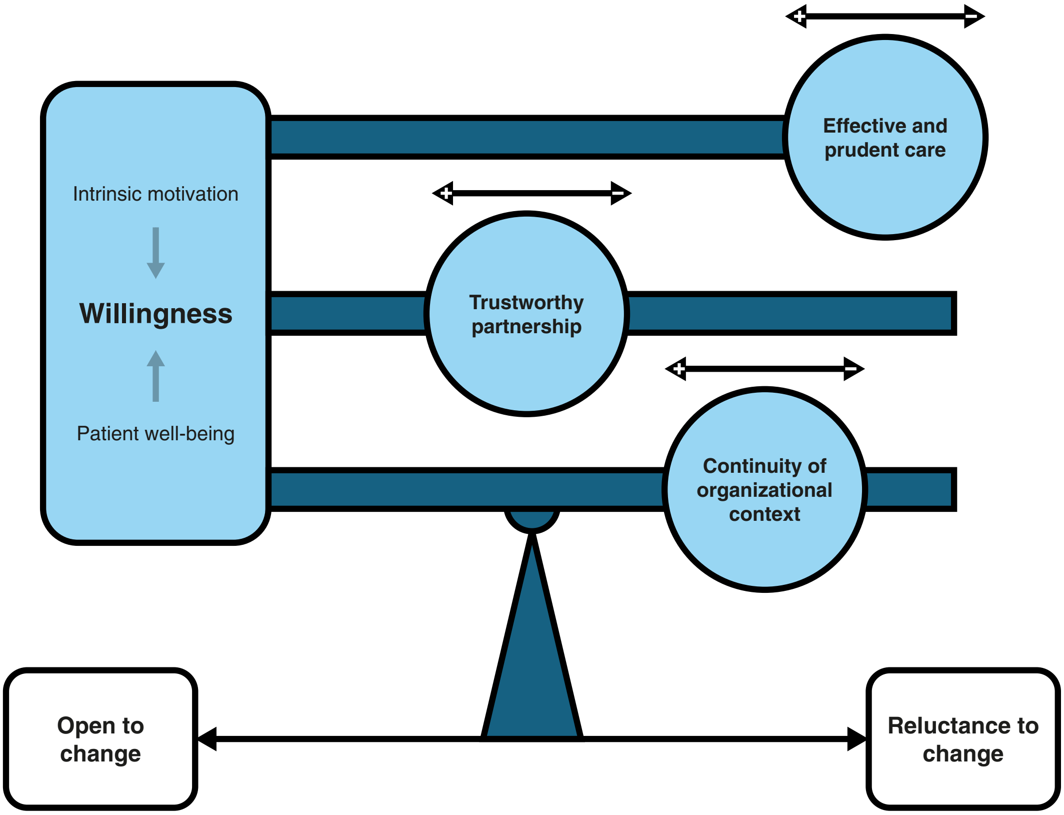

Figure. Conceptual model of innovation adoption in MASLD primary care: a dynamic balance framework. This figure visualizes the overarching and dynamic themes identified in our analysis, using the metaphor of sliding scales. When the scales tip to the left, the GP is inclined to adopt an innovation; when they tip to the right, the GP shows reluctance. The balance reflects how various factors weigh into the decision-making process. On one side, GPs' consistent willingness to change, driven by intrinsic motivation and commitment to patient well-being, serves as a strong enabler of adoption. This willingness is balanced against 3 dynamic and context-sensitive dimensions that can either facilitate or hinder innovation: the perceived alignment with effective and prudent care, the presence of a close and trustworthy partnership with other providers, and the continuity of the organizational context. Each of these themes acts as a fulcrum that can tip the decision toward openness or reluctance, reflecting the complex and multidimensional, ongoing trade-offs in general practice.

Dynamic Theme 1: Primary Care Is Not Merely Hospital Care at a Different Place: The Importance of Effective and Prudent Care

> *One of the things I always tell residents, is to keep patients out of the clutches of a specialist. (Participant 3)*

GPs view themselves as guardians of effective and prudent care, and their reluctance toward certain innovations often stems from differing interpretations with other health-care actors on what exactly this entails. Many described how past innovations have been hindered by discrepancies between perspectives of GPs and specialists on the necessity of innovations (5.1).

Discrepancies often concern the level of evidence required and expected clinical impact. GPs prefer primary care-derived evidence essential before altering practices, rather than extrapolating data from specialist care settings, as participant 6 explained:

> *You have to be able to convince GPs that it is also relevant and effective to do this in a primary care population. And as long as you can't do that, a lot of GPs, who are somewhat conservative by nature, for good reasons, that is also our role in the system, will not take action on it very strongly.*

This reflects the distinct role of GPs in the health-care system. With most patient contacts occurring in primary care, interventions at this level significantly impact overall health-care costs. Subsequently, clinical relevance and efficiency are central to their decisions, as illustrated by participant 9:

> *So first, there is the scientific question: does this screening help? Because we are ex-tre-me-ly critical about screening, right? Since it concerns a very large group.*

This aligns with the GPs' holistic perspective, which prioritizes individual patient well-being over disease-focused care (4.1). GPs consider the full care process, including aspects

Table 2. Interview Excerpts Supporting Identified Themes

Theme	Supporting evidence
Willingness for change	**Intrinsic motivation**<br>“Another key factor is attitude right? Specifically, that GPs are open to change, this differs greatly among them.” (9.1)<br>“But it does motivate to, well, have consultations more often and it is also just informative, so you see that people tend to do it more often… so this learning really does have an effect.” (12.1)<br>**Trade-off**<br>“And you know, in practice you can look at things like: do you have enough people? Do you see the potential? Are you not going to make a loss? Because of course… I mean, we’re entrepreneurs too.” (14.1)
Primary care is not just hospital care at a different place; the importance of effective and prudent care	**Guardians of effective and prudent care**<br>“I now feel that from secondary care it’s more easily said, “that can also be done by the GP,” while I think some people are actually quite complex, and I do want to have said that I really believe that kind of care belongs in secondary care.” (5.1)<br>“So, we’re in consultations with patients all day, and if you ask: who wouldn’t you have wanted to see? The answer is zero, because every patient who sits in front of you has a question, and you want to answer that question. Right? That’s our role.” (4.1)<br>“I do see GPs very much as, what do you call that? Thermometers for this kind of public health developments… So that kind of development small or big, social or medical. You do notice that quite well as a GP.” (11.1)<br>**Uncertainty, gut feeling, and guidelines**<br>“And then you have the 10% who are the teacher’s pets. They’re always ahead of the curve, they know all the guidelines, and they’re also willing to try something new. But they’re the exception. The other 80% just go about their work, some do well, some better, some less so. That’s roughly how you should see it. And yes, there will definitely be doctors who adopt this more quickly. But 80% will just look at the guideline.” (4.2)<br>**Few experience with MASLD and its clinical consequences**<br>“If you can’t present a strong case for it, many people will just think: I’ve got plenty of other things with stronger evidence, good luck with that!” (6.1)
Trustworthy partnership between specialist care and primary care	**Collaboration from the outset**<br>“But in the past, the idea was that the hospital was better, right? Whereas we think: well, it can be done just fine here as well.” (5.2)<br>**Sense of dual partnership, on an equal level**<br>“Usually you will have a project that doesn’t have support then, and if you don’t have support, then that whole project will flop.” (14.2)<br>“You need us, we need you and from that intrinsic motivation it should be: we are partners in crime and how can we help each other?” (4.3)<br>“You have to look at it from a position of equality: how can we best serve the patient? It’s great if we take on certain tasks, but we also need the assurance that a patient can easily return if things don’t go well. So, having a good opportunity for consultation is essential.”(12.2)<br>**Desire for lenient cooperation**<br>“So most general practitioners are so committed to their patients, that they allow that to happen every time.” (2.1)<br>**Informal nature of partnership**<br>“And then the gastroenterologist says: “Can’t you do a calprotectin test?” And this happens quite often of course, that after discussing it a few times, you think: well, I’ll just do it myself now.” (11.2)<br>“Or they see an example somewhere else and think: well, we want that to.” (12.3)<br>**Centralization and the value of the mandate**<br>“And they also have people who can sell that well, well, to the GP practices. And the regional organisation already has a certain goodwill in it, so that’s also convenient. And otherwise, because you have to run it through a separate stream and that’s just not feasible.” (13.1)<br>“My first question is, you talk about implementation: what exactly is implementation?… Because when I sit down with the Ministry of Health, they’re talking about something completely different when it comes to implementation, and when I sit down with researchers, it’s also something entirely different; namely, getting it into the guideline… And the Ministry of Health, yes, what they really want is for people to become healthier, and above all, for it to cost less.” (8.1)

Table 2. Continued

Theme	Supporting evidence
Continuity of organizational context	**Reimbursement**<br>"And it's really not much, just several euros or so per digital consultation, and it does take quite a bit of time, so well, it's basically just cost-covering, but still it is motivating." (12.4)<br>**(Disconnect in perception of) workload**<br>"The extra work you have to do has to be worth the reward. And sometimes, you know… GPs generally have enough, they earn enough. You don't hear many GPs complaining about their income, but you do hear them complain about the workload" (9.2)<br>"So this system changes a little every few years. And that's quite frustrating because you can't make any long-term plans." (7.1)<br>**Low complexity, (due to) integration in existing workflows**<br>"You see, the lab is offering it again in our village, that wasn't the case for a while, but now they're doing it again. Patients no longer have to go to Groningen for it either. Well, now that you mention it, every barrier really is gone… And now it's all just being handed to me on a silver platter." (12.5)<br>**Individualism, pragmatism, and fluidness of feasibility**<br>"You can figure out for yourself how to do it. That's also the fun part of my job." (14.3)

beyond their control, and place high value on patient motivation and engagement. GPs may voice concerns about processes beyond their control, reflecting a broader sense of social responsibility, seeing themselves as indicators of societal disease trends (11.1).

The GP's distinct role also shapes their approach to diagnostic uncertainty. GPs often rely on experience-based gut feeling in decision-making and tend to thrive in such contexts, as participant 14 illustrated:

You know, sometimes, all that us GPs really have are our hands and eyes, and a bag with a few other tools. And that's all we can do.

This ostensibly intuitive approach is contrasted by the high value GPs place on national GP guidelines or regional agreements as a shared reference point within their otherwise individualized practice environment (4.2).

This tension between intuitive practice and guideline reliance may explain why GPs often viewed uncertainty reducing innovations as successful. Working in individualistic settings with autonomous, complex decision-making, GPs rely on guidelines or regional agreements as a supportive framework. In the context of MASLD, care pathways involving NITs may increase their sense of certainty and control.

However, GPs describe urgency for change as arising only when they personally perceive its necessity, as participant 3 noted:

Yes well first, I think there has to be a need! You have to feel that too, that need, otherwise you'll still be stuck in your old…

Of note, GPs do not yet experience MASLD as a pressing issue. Most responded "no" or "maybe a few" when asked if they had referred or managed patients with advanced MASLD fibrosis or cirrhosis. Whether or not this reflects true prevalence, it may contribute to different perceptions of the disease burden between primary and specialist care. In the context of MASLD, this may pose a barrier, as implementation must compete for attention and resources with other priorities across the broad scope of primary care (6.1).

Dynamic Theme 2: Trustworthy Partnership Between Specialist Care and Primary Care

Why that became successful? That's because it was conceived together with GPs and specialists. (Participant 6)

While several factors contribute to strong partnerships, their effectiveness ultimately stems from trust.

GPs consistently noted that earlier innovations succeeded through close transmural collaborations from the outset. When excluded early on, they feel undervalued or disempowered, and innovations often fail to align with daily practice.

Although several participants raised concerns regarding care and workload substitution, most expressed a more nuanced view.

GPs strongly support keeping patients within primary care when appropriate and believe clearly defined problems are best managed there (5.2). The core concern is not task delegation itself but exclusion from preparatory decision-making. Patients are often "dumped," or care tasks shift from specialized to primary care without prior consultation, as described by participant 2:

That's kind of the frustrated answer, but that's kind of what it comes down to. If you get the feeling as a primary care GP that you are carrying it together, then it becomes something very different.

Such lack of communication erodes trust and fosters resistance (14.2). As a result, GPs often instinctually decide whether to engage in innovations, partly based on personal trust in the innovation's initiator, as underlined by participant 10:

And a project like that doesn't really gain traction. You end up with people digging in their heels.

Additionally, GPs view health care as a team effort and treasure partnership (4.3, 12.2).

While they play a central role, this also makes them vulnerable, as they are often held ultimately responsible for the patient. This responsibility can lead them to reluctantly take on tasks passed down by others (2.1), reinforcing their desire for reciprocal and respectful collaboration, as participant 4 expressed:

Have some mercy on the GP! Sometimes they're just thinking: I have no idea either, off you go!

This underscores the central importance of mutual trust, an essential foundation for effective collaboration, whether at the centralized or local level. Early involvement is key to safeguarding professional autonomy and feasibility, but personal relationships are especially important at the local level, as participant 9 described:

Well, I'm truly blessed with a really great hospital, with medical specialists who approach general practitioners in a very respectful and friendly way, as equals. And we quickly developed a kind of personal bond. On top of that, we'd also see each other outside of work; at sports clubs, playing tennis or hockey and so on.

When GPs do deviate from guidelines or local agreements, it is usually after consulting with a specialist or based on a trusted relationship where challenges have been addressed together (11.2, 12.3).

Furthermore, centralization was frequently linked to effective collaboration, tied to organizational capacity, trust, reimbursement, and scale. Innovations are generally more successful when implemented through regional organizations, as they manage insurer funding and are trusted by GPs to coordinate care and innovation (13.1).

This trust is reciprocal; GPs noted that innovations are more effective when insurers allow them to respect their autonomy.

Perceptions of change varied depending on the GP's additional engagements. Those involved nationally focused more on evidence standards, whereas those at local and regional levels, prioritized feasibility. Effective implementation thus requires tailoring strategies to the specific context (8.1). An elegant facilitator for achieving this was suggested by participant 15, who underlined that the most effective way is to engage GPs who convince other GPs: "So if you want someone to take action or implement a care pathway, you also need to speak their language."

Dynamic Theme 3: Continuity of Organizational Context

But the question is: OK, we have reimbursement now, we will have reimbursement in 2 years' time. But if I set something up, I want it to be sustainable. I don't want to say in 3 years' time: well, this is a very good project, but there is no money for it now. (Participant 10)

Alongside identity-related and social context, practical considerations also shape GPs' willingness to adopt innovations, with continuity emerging as a central concern.

Beyond immediate reimbursement, GPs assess whether funding will be sustained long-term. Without this assurance, they are reluctant to invest in new projects or infrastructure.

Even modest reimbursement is a motivator (12.4). As participant 12 explained:

Well that you are appreciated, I think, and that your time is compensated. And even if you are employed, then yes, you just have to do the work and you still get exactly the same amount every month. But still, you do want to see your efforts… reflected, right?

This underscores the earlier mentioned desire for recognition of GPs, which need not be monetary, it may also come as time or staff support.

Innovation feasibility is closely tied to workload reduction, which GPs often cite as essential for successful implementation (9.2). Innovations must address problems GPs actually face, yet many noted a disconnect between their barriers and those perceived by specialists and policymakers:

Sometimes something seems, I think, very simple from the perspective of the hospital, while when you know your patients relatively well, you just know that sometimes a small thing can become more complicated. (Participant 11)

The growing number of NITs increases the need for consistent direction, as GPs tend to resist rapidly shifting policies and strongly value continuity (7.1).

New test strategies must also be simple, as complex procedures do not align with GP workflows. In MASLD, this can be a facilitator, as one GP with NIT experience noted the tests are easy to perform in practice. "Look, of course it's not a huge effort… Every now and then an elevated ALT comes up, and then next time I'll add a FIB4." (Participant 11).

Beyond simplicity, practical accessibility for both patients and GPs also facilitates adoption (12.5). Ideally, tools should be integrated into existing workflows. In the Dutch setting, the structured cardiometabolic care system can serve as a facilitator for implementation of MASLD care pathways, as GPs noted these should be integrated into existing workflows. This is especially important for retesting at-risk patients after several years, as recommended in guidelines. Without such structure, GPs lack the capacity for this proactive recall.

This need for simplicity and integration reflects the broader reality of general practice, where GPs operate in small-scale settings and manage workflows independently. Feasibility is therefore highly individual and sensitive to external factors. This aligns with their pragmatic and holistic approach, as described by participant 10: "I think that if I were to follow the guidelines to the letter, I'd constantly

be in conflict with my patients. Yes, I truly believe that, and it's not because I don't follow the guidelines, but because you're providing personalized care and working within a specific context. And that simply can't be captured in the guidelines."

The participants accentuated that acknowledging this pragmatism in policy and guideline development may serve as a facilitator in the implementation process. Effective innovations should both structure care and accommodate the pragmatic decision-making that defines GP practice (14.3).

Discussion

This qualitative study explored the barriers and facilitators to implementing noninvasive care pathways for MASLD in primary care and provides a deeper understanding of how GPs perceive and might adopt MASLD-related innovations. This study elucidates MASLD-specific implementation dynamics but also offers a conceptual and practical blueprint for similar innovations targeting chronic, underdiagnosed conditions in primary care. GPs showed strong willingness for participation in change, driven by commitment to patient well-being and intrinsic motivation for lifelong learning. However, this is continuously weighed against identity-related, social, and practical factors. Architects of care innovations must recognize that implementation in primary care is not merely logistical and guideline-based but a complex process involving cognitive, attitudinal, motivational, and relational dimensions. Ideally, effective implementation addresses all of these dimensions and is preceded by thorough mapping of local contexts. Without recognizing this complexity, innovation efforts risk limited uptake and sustainability. Implementation strategies should therefore be both evidence-based and context-sensitive.

Previous studies, both quantitative and qualitative, primarily frame barriers as knowledge deficits or organizational issues like reimbursement, implying that education and funding can effectively address these gaps.[9–11,14,28,29] However, our findings indicate that barriers run deeper, rooted in social and cultural factors, including divergent views on quality care, professional identity, and relational dynamics between primary and specialist care. As Wensing and Grol argue, implementation science must address the attitudinal, motivational, and organizational factors shaping clinician behavior, to better reflect health care's real-world complexity.[30] A German study on chronic kidney disease guideline adaptation similarly highlighted the social and practical complexity of decision-making.[7] Notably, our participants did not mention knowledge gaps as past barriers but stressed the importance of making learning relevant, viewing education as a prerequisite, not a barrier for implementation.

This aligns with the findings of the LOCATE intervention, an United Kingdom–based liver disease initiative, where GPs similarly did not view knowledge gaps as barriers, instead emphasizing contextual fit.[31] The focus on knowledge in previous studies may partly reflect participant sampling of those with specific expertise or interest in MASLD, potentially leading to more supportive stances and less critical reflection. It may also stem from a reliance on conceptual or opinion-based input, whereas our interviews were grounded in day-to-day practice, though this distinction cannot be confirmed. The focus on knowledge gaps may further reflect the persistence of the information deficit model in scientific thinking, assuming that inaccurate beliefs stem from lack of knowledge and can be corrected with more or better information.[32] Despite substantial evidence for the contrary, this model has persisted due to its intuitive appeal.[33]

A key implication for practice is the clear divergence in context, perspective, and evidence expectations between primary and specialist care. The hepatologist should recognize that GPs frequently disagree with specialists on the required level of evidence and stress the need for research within primary care settings, engaging GPs in generating and contextualizing evidence. While primary care's role in MASLD management is increasingly recognized, the specific conditions motivating GPs to adopt MASLD-related innovations remain underexplored. This divergence reflects a deeper contrast in clinical reasoning: specialist guidelines often rely on frequentist logic derived from high-prevalence populations, whereas GPs apply Bayesian reasoning, interpreting evidence through prior experience, local prevalence, and individual context. This explains GPs' demand for contextual relevance and their critical stance toward specialist-driven evidence. Ultimately, simply providing information is unlikely to change practice. Bridging the gap between primary and specialist care requires addressing the contextual and cognitive factors that shape how GPs evaluate evidence for each individual patient rather than taking specialist priorities as starting point.

Targeted research into GP-defined implementation prerequisites is urgently needed. In parallel, robust scientific implementation frameworks, such as organizational readiness for change[34] and the Non-adoption, Abandonment, Scale-up, Spread, and Sustainability framework used by Vali et al for NIT implementation for MASLD, should guide interdisciplinary guideline development at both local and central levels.[35] Our study highlights the importance of involving GPs, including those with limited MASLD experience or a critical stance, in future research efforts. Adaptation is more likely to succeed when diverse perspectives, including critical voices, are included.[36]

As discussed, GPs assess innovations based on their quality and impact on both overall health care and individual patients, particularly in relation to clinical guidelines, and they exercise substantial autonomy in deciding which innovations to adopt. Given their broad clinical scope, new initiatives must compete with other priorities and demonstrate sufficient benefit relative to time and resource investment. In the Dutch context, the GPs' strong

reliance on national primary care guidelines issued by the Dutch College of General Practitioners (*Nederlands Huisartsen Genootschap*) may delay uptake of new evidence, hindering MASLD pathway implementation. However, the combination of independent practice and regional GP organizations offers opportunities to implement changes in parallel with guideline revisions. While these contextual features may not be universally transferable to other countries, considering autonomy, guideline reliance, and local organizational structures is essential for effectively advancing MASLD care and can inform tailored approaches in other health-care systems. Furthermore, although not explicitly brought forward by the present study's participants, the presence and active engagement of patient advocacy groups for chronic liver disease may have the potential to positively impact the uptake of MASLD-related innovations.

Although not specific to MASLD, the theme of trustworthy partnership, reflecting the social dynamics of interdisciplinary collaboration, is highly relevant for implementation. GPs, like most people, often rely on intuition or professional instinct when evaluating change, an aspect that should not be overlooked in designing effective implementation strategies.

This study has several strengths. It offers novel insights into barriers and facilitators for integrating preventive hepatology for SLD in primary care, contributing meaningful insights to the field. By focusing on experiential knowledge and combining MASLD-specific and prior innovation experiences, we captured rich, in-depth perspectives on how GPs engage with care innovation and translate these perspectives into a conceptual blueprint that hepatologists can apply themselves.

Our sampling strategy ensured diverse representation across practice types, locations, and roles, enhancing the contextual richness and credibility. Rigorous analysis, following Braun and Clarke's 6-phase framework,[20] alongside iterative data collection and analysis, supported by regular discussions, strengthened analytic depth and dependability. Co-interviewing enabled more dynamic probing and deeper exploration of participant perspectives, enhancing richness of the data.

Our interdisciplinary team, spanning hepatology, internal medicine, general practice, and qualitative research, enhanced understanding and yielded nuanced interpretation, especially concerning Bayesian reasoning and interdisciplinary collaboration.

Finally, our thematic analysis balanced semantic clarity with contextual variation, capturing the complexity and interconnected nature of implementation processes.

Several limitations also need consideration. Limited MASLD-specific experiential knowledge meant most reflections were analogies to other innovations. While this provided valuable insights into implementation dynamic complexity, some interpretations were not grounded in direct experience with MASLD-specific innovations.

As interview-based data, our findings capture perceptions rather than practice, and omit certain practical factors, better addressed through methods like participatory action research.[37]

As qualitative research is not intended to produce generalizable findings, this study aimed for transferability, providing insights into complex contexts to inform understanding and generate hypotheses.

This is particularly relevant given the distinct Dutch context, where GPs act as gatekeepers to specialist care and face unique reimbursement structures. Nonetheless, many social themes and the application of Bayesian reasoning under relative uncertainty are broadly applicable and can enrich understanding diverse health-care systems. Providing detailed contextual information enables readers to applicate our findings to their own setting.

Interviewers N.B. and S.D., both PhD candidates researching noninvasive testing for MASLD, brought a distinct perspective to this GP-focused study, which may have influenced data collection and interpretation. To mitigate this, the multidisciplinary team included GPs in both the study's conceptualization and analysis. Reflexive dialogue and regular team discussions helped identify potential biases and explore differing interpretations.

Although dual interviewing can increase power imbalance in qualitative research, N.B. and S.D., as junior researchers interviewing experienced GPs, did not experience this dynamic.

Conclusion

This study challenges current perspectives on implementing MASLD-related innovations by showing that innovation in primary care is a complex process that extends beyond financial incentives or educational interventions. Although GPs demonstrate strong willingness to adopt new care pathways, there is a constant trade-off with identity-related, relational, and practical factors. Clinical decision-making in primary care follows a distinct logic, characterized by lower disease prevalence, diagnostic uncertainty, and a focus on tangible patient impact.

In MASLD, complexity is further heightened by limited experiential knowledge, low perceived urgency, and skepticism toward the current evidence base. GPs assess innovations pragmatically through a Bayesian lens, balancing expected benefit against disruption to continuity, time investment, and workload, while placing high value on trustworthy partnerships. Education remains pivotal, but should be introduced just-in-time.

Implementation strategies that acknowledge this reasoning, by underscoring contextual fit, practical feasibility, and collaboration, are more likely to succeed. Sustainable implementation requires moving beyond awareness and reimbursement toward codesigned, workflow-integrated, and practice-based approaches that resonate with GPs' clinical reasoning, professional identity, and local context.

Supplementary Materials

Material associated with this article can be found, in the online version, at https://doi:10.1016/j.gastha.2026.100991.

References

1. Barrow JM, Annamaraju P. Change management in health care. In: Statpearls. Treasure Island (FL): StatPearls Publishing, 2025.
2. Younossi ZM, Golabi P, Paik JM, et al. The global epidemiology of nonalcoholic fatty liver disease (NAFLD) and nonalcoholic steatohepatitis (NASH): a systematic review. Hepatology 2023;77:1335–1347.
3. Younossi ZM, Razavi H, Sherman M, et al. Addressing the high and rising global burden of metabolic dysfunction-associated steatotic liver disease (MASLD) and metabolic dysfunction-associated steatohepatitis (MASH): from the growing prevalence to payors' perspective. Aliment Pharmacol Ther 2025;61:1467–1478.
4. Rinella ME, Neuschwander-Tetri BA, Siddiqui MS, et al. AASLD Practice Guidance on the clinical assessment and management of nonalcoholic fatty liver disease. Hepatology 2023;77:1797–1835.
5. European Association for the Study of the Liver (EASL); , European Association for the Study of Diabetes (EASD); European Association for the Study of Obesity (EASO). EASL-EASD-EASO Clinical Practice Guidelines on the management of metabolic dysfunction-associated steatotic liver disease (MASLD). J Hepatol 2024;17:374–444.
6. Anstee QM, Hallsworth K, Lynch N, et al. Real-world management of non-alcoholic steatohepatitis differs from clinical practice guideline recommendations and across regions. JHEP Rep 2022;4:100411.
7. Laker K, Bothe T, Ebert N, et al. Guidelines or mindlines? - Implementing a new CKD guideline in German primary care. BMC Prim Care 2024;25:344.
8. Fathy C, Patel S, Sternberg P Jr, et al. Disparities in adherence to screening guidelines for diabetic retinopathy in the United States: a comprehensive review and guide for future directions. Semin Ophthalmol 2016; 31:364–377.
9. Gracen L, Hayward KL, Aikebuse M, et al. An exploration of barriers and facilitators to implementing a nonalcoholic fatty liver disease pathway for people with type 2 diabetes in primary care. Diabet Med 2022;39: e14799.
10. Standing HC, Jarvis H, Orr J, et al. GPs' experiences and perceptions of early detection of liver disease: a qualitative study in primary care. Br J Gen Pract 2018; 68:e743–e749.
11. Tsochatzis EA, Valenti L, Thiele M, et al. Use of non-invasive diagnostic tools for metabolic dysfunction-associated steatohepatitis: a qualitative exploration of challenges and barriers. Liver Int 2024;44:1990–2001.
12. Grol R, Wensing M. What drives change? Barriers to and incentives for achieving evidence-based practice. Med J Aust 2004;180:S57–S60.
13. Wieringa S, Dreesens D, Forland F, et al. Different knowledge, different styles of reasoning: a challenge for guideline development. BMJ Evid Based Med 2018; 23:87–91.
14. Younossi ZM, Ong JP, Takahashi H, et al. A global survey of physicians knowledge about nonalcoholic fatty liver disease. Clin Gastroenterol Hepatol 2022; 20:e1456–e1468.
15. Bours MJL. Bayes' rule in diagnosis. J Clin Epidemiol 2021;131:158–160.
16. Lazarus JV, Mark HE, Anstee QM, et al. Advancing the global public health agenda for NAFLD: a consensus statement. Nat Rev Gastroenterol Hepatol 2022; 19:60–78.
17. Lazarus JV, Mark HE, Allen AM, et al. A global research priority agenda to advance public health responses to fatty liver disease. J Hepatol 2023;79:618–634.
18. Kremers MNT, Nanayakkara PWB, Levi M, et al. Strengths and weaknesses of the acute care systems in the United Kingdom and the Netherlands: what can we learn from each other? BMC Emerg Med 2019;19:40.
19. den Engelsen C, Soedamah-Muthu SS, Oosterheert NJA, et al. Improved care of type 2 diabetes patients as a result of the introduction of a practice nurse: 2003–2007. Prim Care Diabetes 2009;3:165–171.
20. Braun V, Clarke V. Thematic analysis: a practical guide. Thousand Oaks, CA: SAGE, 2022.
21. Sandelowski M. Sample size in qualitative research. Res Nurs Health 1995;18:179–183.
22. Ratziu V, Anstee QM, Wong VW, et al. An international survey on patterns of practice in NAFLD and expectations for therapies-the POP-NEXT project. Hepatology 2022;76:1766–1777.
23. Velardo S, Elliott S. Co-Interviewing in qualitative social research: prospects, merits and considerations. Int J Qual Methods 2021;20:160940692110549.
24. LaDonna KA, Artino AR Jr, Balmer DF. Beyond the guise of saturation: rigor and qualitative interview data. J Grad Med Educ 2021;13:607–611.
25. Malterud K, Siersma VD, Guassora AD. Sample size in qualitative interview studies: guided by information power. Qual Health Res 2016;26:1753–1760.
26. Creswell JW, Poth CN. Qualitative inquiry and research design: choosing among five approaches. Sage Publications, 2016.
27. Stenfors T, Kajamaa A, Bennett D. How to … assess the quality of qualitative research. Clin Teach 2020; 17:596–599.
28. Lazure P, Tomlinson JW, Kowdley KV, et al. Clinical practice gaps and challenges in non-alcoholic

steatohepatitis care: an international physician needs assessment. Liver Int 2022;42:1772–1782.
29. Driessen S, de Jong VD, van Son KC, et al. A global survey of health care workers' awareness of non-alcoholic fatty liver disease: the AwareNASH survey. United European Gastroenterol J 2023;11:654–662.
30. Wensing M, Grol R. Knowledge translation in health: how implementation science could contribute more. BMC Med 2019;17:88.
31. Reinson T, Bradbury K, Moore M, et al. Healthcare practitioners' experiences of an intervention to detect and treat patients with liver disease (the LOCATE intervention): a qualitative process evaluation. BMJ Open 2019;9:e028591.
32. Simis MJ, Madden H, Cacciatore MA, et al. The lure of rationality: why does the deficit model persist in science communication? Public Underst Sci 2016;25:400–414.
33. Sturgis P, Allum N. Science in society: re-evaluating the deficit model of public attitudes. Public Underst Sci 2004;13:55–74.
34. Weiner BJ. A theory of organizational readiness for change. Implement Sci 2009;4:67.
35. Vali Y, Eijk R, Hicks T, et al. Clinicians' perspectives on barriers and facilitators for the adoption of non-invasive liver tests for NAFLD: a mixed-method study. J Clin Med 2022;11:2707.
36. Armenakis AA, Harris SG. Reflections: our journey in organizational change research and practice. J Change Manag 2009;9:127–142.
37. Kindon S, Pain R, Kesby M. In: Participatory action research: origins, approaches and methods. Participatory action research approaches and methods. London, UK: Routledge, 2007:35–44.

Received October 6, 2025. Accepted April 27, 2026.

Correspondence:
Address correspondence to: Stan Driessen, MD, PhD, Department of Vascular Medicine and Hepatology, Amsterdam University Medical Centre, Meibergdreef 9, Amsterdam 1105AZ, the Netherlands. e-mail: s.driessen2@amsterdamumc.nl.

Acknowledgments:
We thank all participating general practitioners for their valuable insights. We also thank David Brophy for providing assistance in translating all selected quotes into English. We thank Eric Moll van Charante for his helpful input during the study design phase.

Authors' Contributions:
Stan Driessen and Marianne C. Mak-Van der Vossen: Conceptualization. Stan Driessen, Leonard (Niels) D. Broekman, Anne de la Croix, Marianne C. Mak-Van der Vossen, and Otto R. Maarsingh: Methodology. Stan Driessen, Leonard (Niels) D. Broekman, Anne de la Croix, Marianne C. Mak-Van der Vossen, Maarten E. Tushuizen, Otto R. Maarsingh, and Adriaan (Onno) G. Holleboom: Validation. Stan Driessen, Leonard (Niels) D. Broekman, Anne de la Croix, Marianne C. Mak-Van der Vossen, Maarten E. Tushuizen, Otto R. Maarsingh, and Adriaan (Onno) G. Holleboom: Formal analysis. Stan Driessen and Leonard (Niels) D. Broekman: Investigation. Stan Driessen and Leonard (Niels) D. Broekman: Data curation. Stan Driessen and Leonard (Niels) D. Broekman: Writing - original draft. Stan Driessen, Leonard (Niels) D. Broekman, Anne de la Croix, Marianne C. Mak-Van der Vossen, Maarten E. Tushuizen, Otto R. Maarsingh, and Adriaan (Onno) G. Holleboom: Writing - review and editing. Stan Driessen and Leonard (Niels) D. Broekman: Visualization. Anne de la Croix, Marianne C. Mak-Van der Vossen, Maarten E. Tushuizen, Otto R. Maarsingh, and Adriaan (Onno) G. Holleboom: Supervision. Stan Driessen, Leonard (Niels) D. Broekman, Anne de la Croix, Marianne C. Mak-Van der Vossen, and ` (Onno) G. Holleboom: Project administration.

Conflicts of Interest:
The authors disclose no conflicts.

Funding:
This study received the following grant support: MLDS NLA2 grant, WO-21-06 and Innovative Health Initiative Joint Undertaking (IHI JU), grant agreement No. 101132946. The funders had no role in design, conduct, and reporting of the study.

Ethical Statement:
The study followed the Declaration of Helsinki, was approved by the Amsterdam UMC Ethics Committee and exempted from further review (2025.0321), followed institutional and General Data Protection Regulation guidelines, and obtained audio-recorded informed consent. Data were deidentified and handled per Amsterdam UMC Public Health research code.

Data Transparency Statement:
The code tree and audit trail are available to other investigators upon reasonable request by contacting the corresponding author. Interview recordings and transcripts are not publicly available due to participant privacy considerations.

Reporting Guidelines:
The Standards for Reporting Qualitative Research (SRQR) Checklist was followed and is included as separate supplemental.

Gastro Hep Advances, Volume 5

Supplemental information

Beyond Awareness: A Qualitative Assessment of Barriers and Facilitators to Implementing Metabolic Dysfunction–Associated Steatotic Liver Disease Care Pathways in Primary Care

Stan Driessen, Leonard (Niels) D. Broekman, Anne de la Croix, Marianne C. Mak-Van der Vossen, Maarten E. Tushuizen, Otto R. Maarsingh, and Adriaan (Onno) G. Holleboom

Supplemental Material

Beyond awareness: a qualitative assessment of barriers and facilitators to implementing MASLD care pathways in primary care

Shared first authors: Stan Driessen, Leonard (Niels) D. Broekman

Table of contents:

Supplemental material 1. Description of Dutch healthcare system	P. 2
Supplemental material 2. Final interview guide, translated to English	P. 3
Supplemental material 3. Final interview guide, original Dutch version	P. 6
Supplemental material 4. Complete description of how analytic sufficiency was reached	P. 9

Supplemental material 1. Description of Dutch healthcare system

The Dutch healthcare system can be defined as a system of regulated market competition consisting of three distinct markets.(1) First, the health insurance market between patient and insurer, second, the health care purchasing market between insurer and provider, and third, the health care provision market between provider and patient. All Dutch inhabitants are obliged to arrange a basic insurance plan with one of the health insurance companies on the insurer's market. All health insurers are legally obliged to accept any applicant for their basic insurance package, ensuring universal access regardless of health status. Next to the individual premiums for mandatory basic health insurance, the Dutch healthcare system is primarily funded through employer and government contributions. Basic insurance plans cover all consultations in primary care and keep out-of-pocket costs low. GP compensation is a mix of capitation and fee-for-service payments. In the Dutch system, GPs are the first point of contact for most medical concerns and management of several chronic conditions e.g. type 2 diabetes mellitus and COPD as well as cardiovascular risk management. Furthermore, GPs function as gatekeepers providing personalised referrals to emergency departments or outpatient clinics in regional hospitals, university hospitals, or independent treatment centres. For most outpatient, specialist care, patients pay a maximum out-of-pocket deductible per year. Health insurers contract with hospitals and independent treatment centres, and medical specialists are generally organised within partnerships who are on their turn contracted by the hospitals. Hospitals are predominantly private, independently managed non-profit entities who operate under national regulations. Within this system, and through freedom of choice for patients, hospitals are expected to compete on quality and efficiency, whereas primary care is exempted from these incentives. The Dutch government has a regulatory role, overseeing coverage decisions and managing risk equalisation measures that compensate insurers for taking on high-risk patients.

Supplemental material 2. Final interview guide, translated to English

<table>
<tr><th colspan="3">BARRIERS AND FACILITATORS IN IMPLEMENTING CARE PATHWAYS FOR MASLD IN DUTCH PRIMARY CARE</th></tr>
<tr><td rowspan="3">Background Information</td><td>Interviewers</td><td>S. Driessen
L.D. Broekman</td></tr>
<tr><td>Supervisors</td><td>A. de la Croix
M.C. Mak-van der Vossen
M.E. Tushuizen
O.R. Maarsingh
A.G. Holleboom</td></tr>
<tr><td>Organisation</td><td>Vascular Medicine & General pratice/Family medicine, Amsterdam UMC</td></tr>
<tr><td>Introduction</td><td colspan="2">• First of all, I want to thank you for taking the time to participate in this interview.
• Do you prefer to be addressed formally or informally?
• I’d like to give you some background about this research project, and then explain how I plan to conduct this interview.
• If you have any questions before the interview starts, you can ask them after the explanation.</td></tr>
<tr><td>Background</td><td colspan="2">• Currently, there is global interest in implementing care pathways for diagnosing liver fibrosis due to metabolic dysfunction-associated steatotic liver disease (MASLD), previously known as non-alcoholic fatty liver disease (NAFLD). In the Netherlands, there is no standardised testing for liver fibrosis within a care pathway.
• A Dutch multicenter study is currently underway investigating the effectiveness of these pathways across different levels of care. However, it is equally important to assess whether these pathways are also desirable and feasible in daily practice.</td></tr>
<tr><td>Research aim</td><td colspan="2">• To explore the general practitioner’s perspective on perceived barriers and facilitators for implementing MASLD fibrosis care pathways.</td></tr>
<tr><td>Privacy</td><td colspan="2">• The data will be used for publication in a scientific article.
• Your personal information will only be accessed by our research team at Amsterdam UMC.
• In the article, personal data will be pseudonymised. The results will not be traceable back to you.</td></tr>
<tr><td>Duration</td><td colspan="2">• This interview will take approximately 30 to 40 minutes.</td></tr>
<tr><td>Additional questions</td><td colspan="2">• Do you have any additional questions?</td></tr>
</table>

**Recording**	• This interview will be audio recorded<br>• Legally, I am required to ask for your permission to do so.<br>• START RECORDING<br>• Do I have your permission to record this interview?
**INTRODUCTORY QUESTION**	
**Interviewee Background**	• Can you briefly tell me who you are, where you work, and what kind of general practice you work in?<br>• How many years have you been working as a GP?<br>• Do you have a specific area of expertise?
**Domain 1:**<br>*Experiences with MASLD in Daily Practice*	Can you describe how you approach MASLD in your daily practice?<br><br>• Have you ever referred a patient for hepatic steatosis?<br>• Do you currently have a patient with MASH-cirrhosis?
**Domain 2:**<br>*Other Innovations in General Practice*	What are your experiences with previous innovations in primary care? What facilitated them, and what made them difficult?<br><br>• **Can you give an example?**<br>• Can you draw a parallel with hepatic steatosis?<br>• What has your experience been with communication from the hospital regarding this care (or previous implementations)?<br>• What aspects are often overlooked?<br>• What makes GPs enthusiastic about certain changes?
**CLOSING QUESTION**	
**Summary**	We are nearing the end of this interview. As a final question, could you briefly summarise:<br>• The most important facilitators for implementing these non-invasive MASLD tests in your practice, from your perspective?<br>• The most important barriers to implementing these non-invasive MASLD tests in your practice, from your perspective?
**Final Remark**	• Would you like to add anything that we haven't discussed yet?
**CLOSING THE INTERVIEW**	
**Conclusion**	• That brings us to the end of this interview.
**Thanks**	• I sincerely want to thank you for your time and participation. I really enjoyed this conversation and I hope you did too.
**Next Steps**	• This interview will be transcribed verbatim.

	• Afterwards, I will send you a one-page summary for review. This gives you the opportunity to add anything that may have been missed.<br>• The main findings from this interview will be compared with the main findings from other interviews.<br>• After analysis, the results will be incorporated into a scientific article.<br>• Would you like to receive a message when the paper is published?<br>• Do you have any further questions?<br>• STOP RECORDING

Supplemental material 3. Final interview guide, original Dutch version

<table>
<tr><th colspan="3">VALKUILEN EN BEVORDERAARS IN HET IMPLEMENTEREN VAN ZORGPADEN VOOR MASLD IN DE NEDERLANDSE EERSTE LIJN ZORG</th></tr>
<tr><td rowspan="3">Achtergrond informatie</td><td>Interviewers</td><td>S. Driessen
L.D. Broekman</td></tr>
<tr><td>Supervisors</td><td>A. de la Croix
M.C. Mak-van der Vossen
M.E. Tushuizen
O.R. Maarsingh
A.G. Holleboom</td></tr>
<tr><td>Organisatie</td><td>Vasculaire geneeskunde & Huisartsgeneeskunde, Amsterdam UMC</td></tr>
<tr><td>Introductie</td><td colspan="2">• Eerst en vooral wil ik u bedanken voor vrijmaken van uw tijd om deel te nemen aan dit interview.
• Heeft u liever dat ik vousvoyeer of tutoyeer?
• Ik wil u wat achtergrondinformatie geven over dit onderzoeksproject. Daarna zal ik uitleg geven over de manier waarop ik dit interview wil afnemen.
• Als u vragen hebt voordat het interview begint, kunt u ze na de uitleg stellen.</td></tr>
<tr><td>Achtergrond</td><td colspan="2">• Momenteel is er wereldwijd veel belangstelling voor het implementeren van zorgpaden voor diagnostiek naar leverfibrose als gevolg van metabolic dysfunction associated steatotic liver disease (MASLD), voorheen non-alcoholic fatty liver disease (NAFLD). In Nederland wordt momenteel nog niet standaard getest op leverfibrose in een zorgpad.
• Er loopt momenteel een Nederlandse multicenter studie die de effectiviteit van deze zorgpaden onderzoekt in meerdere lijnen van zorg. Echter is het ook van belang om te weten of de zorgpaden ook gewenst zijn en uitvoerbaar zijn in de praktijk.</td></tr>
<tr><td>Doel onderzoek</td><td colspan="2">• In kaart brengen van perspectief van de huisarts op gepercipieerde barrières en facilitatoren voor zorgpadimplementatie voor MASLD-fibrose.</td></tr>
<tr><td>Privacy</td><td colspan="2">• De gegevens worden gebruikt voor publicatie in een artikel.
• Uw persoonlijke gegevens worden alleen ingezien door ons onderzoeksteam in het Amsterdam UMC.
• In het artikel worden persoonlijke gegevens gepseudonimiseerd. De resultaten van dit onderzoek zullen dus niet tot u herleidbaar zijn.</td></tr>
</table>

**Duur**	• Dit interview duurt ongeveer 30 tot 40 minuten.
**Aanvullende vragen**	• Heeft u nog aanvullende vragen?
**Opname**	• Van dit interview wordt een geluidsopname gemaakt<br>• Het is wettelijk verplicht dat ik toestemming vraag om dit te doen.<br>• START OPNAME<br>• Heb ik uw toestemming om dit interview op te nemen?
**INLEIDENDE VRAAG**	
**Achtergrond geïnterviewde**	• Kunt u mij in het kort vertellen wie u bent, waar u werkt en in wat voor soort huisartspraktijk u werkt?<br>• Hoeveel jaar werkt u al als huisarts?<br>• Heeft u een specifiek expertisegebied?
**Domein 1:**<br>*Ervaringen met MASLD in de dagelijkse praktijk*	Kunt u vertellen hoe u omgaat met MASLD in uw dagelijkse praktijk?<br><br>• Heeft u wel eens een patiënt verwezen voor leversteatose?<br>• Heeft u een patiënt in de praktijk met MASH-cirrose?
**Domein 2:**<br>*Andere innovaties*	Wat zijn uw ervaringen met eerdere innovaties in de huisartsenzorg? Wat heeft dit gefaciliteerd, wat maakte het lastig?<br><br>• **Kunt u daar een voorbeeld van geven?**<br>• Kunt u een parallel trekken met leversteatose?<br>• Hoe is de ervaring met communicatie vanuit het ziekenhuis omtrent deze zorg (of eerdere implementaties)?<br>• Welke zaken worden meestal over het hoofd gezien?<br>• Wat maakt dat huisartsen ergens enthousiast voor worden?
**AFSLUITENDE VRAAG**	
**Samenvatting**	We naderen het einde van dit interview. Als laatste vraag: kunt u kort samenvatten:<br>• De belangrijkste facilitators voor de implementatie van deze niet-invasieve MASLD-testen in uw praktijk vanuit uw perspectief?<br>• De belangrijkste barrières voor de implementatie van deze niet-invasieve MASLD-testen in uw praktijk vanuit uw perspectief?
**Slotopmerking**	• Wilt u nog iets toevoegen dat we nog niet hebben besproken?
**AFSLUITING INTERVIEW**	
**Afsluiting**	• Daarmee is dit interview tot het einde gekomen

**Dank**	• Ik wil u oprecht bedanken voor uw tijd en deelname aan dit interview. Ik vond het erg leuk om dit gesprek met u te hebben en ik hoop dat u het ook zo hebt ervaren.
**Vervolgstappen**	• Dit interview zal woordelijk worden getranscribeerd.<br>• Daarna stuur ik een samenvatting van ongeveer één pagina ter controle. Dit geeft u de mogelijkheid om iets toe te voegen aan wat er is besproken.<br>• De belangrijkste resultaten van dit interview worden vergeleken met de belangrijkste resultaten van de andere interviews.<br>• Na analyse zullen de resultaten verwerkt worden in een nog te publiceren artikel.<br>• Wilt u een bericht ontvangen als er een paper gepubliceerd gaat worden?<br>• Heeft u verder nog vragen?<br>• STOP OPNAME

Supplemental material 4. Complete description of how analytic sufficiency was reached

We determined the end of data collection based on the methodologically grounded principle of analytic sufficiency, which offers a more nuanced and robust alternative to the increasingly contested concept of data saturation. Following the iterative nature of thematic analysis, data collection and analysis were conducted concurrently. As interviews progressed, we observed decreasing novelty in the coding process, and the core themes became increasingly well-defined and richly supported.

We applied the concept of information power, considering the study aim, sample specificity, quality of dialogue, and the strength of the established themes. While our sample was diverse in terms of practice type, MASLD experience, and involvement in innovation, the relatively narrow study aim, in combination with the richness of the data due to co-interviewing, led to high information power.

Analytic sufficiency was confirmed when no new relevant dimensions emerged and existing themes were internally coherent. The four themes were fully disentangled from the data after interview 11. The remaining four interviews mostly added depth to the themes and their interrelations. The broad context of theme “Trustworthy partnership between specialist care and primary care” and theme “Continuity of organisational context”, indicate that it would have been possible to possibly gain more in-depth insights with more interviews. Given the MASLD-specific research question, we decided after team discussions that this would not have added additional value to this specific context and we concluded that additional interviews would likely lead to redundancy rather than added insight. This decision was reached collaboratively by the entire research team after 15 interviews. Member checking was not conducted, as participants’ accounts were concrete and closely linked to their professional experiences, leaving little scope for reinterpretation. While member checking is well-established in qualitative research, it is not universally necessary or desirable, especially in contexts like qualitative health studies where clear, descriptive data already robustly support thematic findings.(2)

References

1. Misser NS, Versendaal PhD J, Methorst M, Stork B. Dutch Healthcare: An Overview and Application. 2014.
2. Thomas DR. Feedback from research participants: are member checks useful in qualitative research? Qualitative Research in Psychology 2017;14:23-41.

COREQ (COnsolidated criteria for REporting Qualitative research) Checklist

A checklist of items that should be included in reports of qualitative research. You must report the page number in your manuscript where you consider each of the items listed in this checklist. If you have not included this information, either revise your manuscript accordingly before submitting or note N/A.

Topic	Item No.	Guide Questions/Description	Reported on Page No.
**Domain 1: Research team and reflexivity**			
Personal characteristics			
Interviewer/facilitator	1	Which author/s conducted the interview or focus group?	11
Credentials	2	What were the researcher's credentials? E.g. PhD, MD	9
Occupation	3	What was their occupation at the time of the study?	9
Gender	4	Was the researcher male or female?	x
Experience and training	5	What experience or training did the researcher have?	9
Relationship with participants			
Relationship established	6	Was a relationship established prior to study commencement?	10
Participant knowledge of the interviewer	7	What did the participants know about the researcher? e.g. personal goals, reasons for doing the research	supplemental
Interviewer characteristics	8	What characteristics were reported about the inter viewer/facilitator? e.g. Bias, assumptions, reasons and interests in the research topic	9, 11, 25
**Domain 2: Study design**			
Theoretical framework			
Methodological orientation and Theory	9	What methodological orientation was stated to underpin the study? e.g. grounded theory, discourse analysis, ethnography, phenomenology, content analysis	9, 11
Participant selection			
Sampling	10	How were participants selected? e.g. purposive, convenience, consecutive, snowball	10
Method of approach	11	How were participants approached? e.g. face-to-face, telephone, mail, email	10
Sample size	12	How many participants were in the study?	11
Non-participation	13	How many people refused to participate or dropped out? Reasons?	10, 11
Setting			
Setting of data collection	14	Where was the data collected? e.g. home, clinic, workplace	10
Presence of non-participants	15	Was anyone else present besides the participants and researchers?	11
Description of sample	16	What are the important characteristics of the sample? e.g. demographic data, date	13
Data collection			
Interview guide	17	Were questions, prompts, guides provided by the authors? Was it pilot tested?	10, supplemen
Repeat interviews	18	Were repeat inter views carried out? If yes, how many?	11, supplemer
Audio/visual recording	19	Did the research use audio or visual recording to collect the data?	11
Field notes	20	Were field notes made during and/or after the inter view or focus group?	11
Duration	21	What was the duration of the inter views or focus group?	11
Data saturation	22	Was data saturation discussed?	11, supplemen
Transcripts returned	23	Were transcripts returned to participants for comment and/or	supplemental

Topic	Item No.	Guide Questions/Description	Reported on Page No.
		correction?	
**Domain 3: analysis and findings**			
Data analysis			
Number of data coders	24	How many data coders coded the data?	11,12
Description of the coding tree	25	Did authors provide a description of the coding tree?	11
Derivation of themes	26	Were themes identified in advance or derived from the data?	9, 11
Software	27	What software, if applicable, was used to manage the data?	11
Participant checking	28	Did participants provide feedback on the findings?	supplemental
Reporting			
Quotations presented	29	Were participant quotations presented to illustrate the themes/findings? Was each quotation identified? e.g. participant number	13-20
Data and findings consistent	30	Was there consistency between the data presented and the findings?	13-20, 21-23
Clarity of major themes	31	Were major themes clearly presented in the findings?	13
Clarity of minor themes	32	Is there a description of diverse cases or discussion of minor themes?	13-20

Developed from: Tong A, Sainsbury P, Craig J. Consolidated criteria for reporting qualitative research (COREQ): a 32-item checklist for interviews and focus groups. *International Journal for Quality in Health Care*. 2007. Volume 19, Number 6: pp. 349 – 357

Once you have completed this checklist, please save a copy and upload it as part of your submission. DO NOT include this checklist as part of the main manuscript document. It must be uploaded as a separate file.
